# Semi-Supervised Multi-View Learning for Gene Network Reconstruction

**DOI:** 10.1371/journal.pone.0144031

**Published:** 2015-12-07

**Authors:** Michelangelo Ceci, Gianvito Pio, Vladimir Kuzmanovski, Sašo Džeroski

**Affiliations:** 1 Dept. of Computer Science, University of Bari Aldo Moro, Via Orabona 4, 70125 Bari, Italy; 2 Dept. of Knowledge Technologies, Jožef Stefan Institute, Jamova 39, 1000 Ljubljana, Slovenia; 3 Jožef Stefan International Postgraduate School, Jamova 39, 1000 Ljubljana, Slovenia; Plymouth University, UNITED KINGDOM

## Abstract

The task of gene regulatory network reconstruction from high-throughput data is receiving increasing attention in recent years. As a consequence, many inference methods for solving this task have been proposed in the literature. It has been recently observed, however, that no single inference method performs optimally across all datasets. It has also been shown that the integration of predictions from multiple inference methods is more robust and shows high performance across diverse datasets. Inspired by this research, in this paper, we propose a machine learning solution which *learns to combine* predictions from multiple inference methods. While this approach adds additional complexity to the inference process, we expect it would also carry substantial benefits. These would come from the automatic adaptation to patterns on the outputs of individual inference methods, so that it is possible to identify regulatory interactions more reliably when these patterns occur. This article demonstrates the benefits (in terms of accuracy of the reconstructed networks) of the proposed method, which exploits an iterative, semi-supervised ensemble-based algorithm. The algorithm learns to combine the interactions predicted by many different inference methods in the multi-view learning setting. The empirical evaluation of the proposed algorithm on a prokaryotic model organism (E. coli) and on a eukaryotic model organism (S. cerevisiae) clearly shows improved performance over the state of the art methods. The results indicate that gene regulatory network reconstruction for the real datasets is more difficult for S. cerevisiae than for E. coli. The software, all the datasets used in the experiments and all the results are available for download at the following link: http://figshare.com/articles/Semi_supervised_Multi_View_Learning_for_Gene_Network_Reconstruction/1604827.

## Introduction

In the last decade, the developments in systems biology have resulted in an improved understanding of the working mechanisms in an organism, which is described as a complex and dynamical system with multiple levels of regulation. Such levels of regulation are typically represented via biological networks that model the complex interactions which occur among different components in a cell. In particular, regulations modeled by such biological networks include the control of transcription into mRNA (messenger RNA) and the translation into protein macromolecules, which are the main building blocks of the elementary unit of life [1, 2]. Such biological networks are typically referred to as Gene-Regulatory Networks (GRNs). In a GRN, nodes represent molecular entities, such as transcription factors (TFs), proteins and metabolites, whereas edges represent interactions, such as protein-protein and protein-DNA interactions.

Identifying the structure of GRNs helps in the biological understanding of disease mechanisms and raises possibilities for better medical/clinical care by improving diagnostics, prognostics and treatment [3]. It comprises the identification of pairwise interactions between molecules (nodes in a network) that participate in the same biological processes or that perform together specific biological functions that shape a system’s behavior and function [4]. The structure of the network can be elucidated experimentally by using ChIP-chip or ChIP-sequencing [5], bacterial one-hybrid system [6] or protein-binding microarrays [7], which are technically and often financially demanding [1]. Alternatively, measuring the dynamic response of transcription and translation within a cell can provide robust information about the GRN under consideration. The information about the GRN comes from microarray experiments perturbating and stressing genes that produce highly resolved time-series and steady-state measurements of transcript levels. The availability of such information offers a way to infer the topology of the network via data-driven approaches.

A comprehensive review of the literature shows the existence of a wide range of data-driven approaches for inferring GRNs (this task is also referred to as “reverse-engineering” or simply “network reconstruction”). In particular, there are some review articles covering this field with well-structured overviews of the general ideas behind the inference process [8–11]. Existing methods differ from each other significantly and the ideas behind each method of GRN reconstruction generally do not belong to a single theory, but rather come from distinct classes of statistical/mathematical methods and information/machine learning theory. The different network reconstruction methods are evaluated and compared within the DREAM (Dialogue for Reverse Engineering Assessments and Methods) series of challenges, which has a significant impact on the development of this field. In this series, researchers present their newly developed approaches for inferring GRNs and compare them to existing ones on a set of benchmark problems of network reconstruction. In a follow-up study [12], the authors empirically prove that combining, by averaging ranks, prediction scores of all the presented methods (“Wisdom of crowds”) can result in a more accurate network reconstruction. A more sophisticated solution for combining the output of several methods has been proposed by Hase et al. [13]. In such work, the predictions of each method are ranked according to their scores and the “combined rank” of each interaction is computed by taking the *k*-th highest rank among all the considered methods, where *k* is an input parameter.

Following this main stream of research, our study focuses on a machine learning-based combination of methods’ outcomes in order to further improve the identification of relationships among the nodes in a reconstructed network. In particular, the solution we propose is to build a (possibly stronger) predictive model by considering as input features the scores returned by several algorithms for gene network reconstruction (henceforth “base methods”), resorting to a solution which is borrowed from studies in meta-learning [14]. However, the application of existing meta-learning approaches is not trivial since the task at hand demands tailored solutions taking into account several specific issues: *i)* Considering as many as possible base methods to increase the final prediction accuracy can lead to the construction of a highly redundant set of input features. This phenomenon is particularly evident when the considered base methods (or groups of them) are based on the same main principle (e.g., correlation between expressions of genes). *ii)* The set of known regulatory interactions to exploit for building the predictive model consists of very few examples of interactions (or even none). Moreover, their labels are generally only positive (i.e., existing regulatory interactions), whereas no examples labeled as negative (i.e., non-existing regulatory interactions) are usually available. *iii)* The number of interactions for which the label is known is strongly *imbalanced* with respect to the number of interactions for which the label is unknown. Classical machine learning tools only exploit labeled examples when building a predictive model, disregarding information conveyed by unlabeled examples.

Concerning *i)*, the most straightforward solution is the application of feature selection algorithms or the adoption of classifiers which inherently take into account the possible redundancy among the features. However, in this case, despite the redundancy, the number of features is limited (by the number of considered base methods) and we want to exploit all the features, even if they are highly correlated with the others. To this aim, we consider the multi-view learning framework, a variant of the co-training framework [15]. The basic idea is to learn classifiers on different views (typically, different feature sets) of the same dataset and iteratively use predictions of one classifier as training instances for the other classifiers.

According to Blum and Mitchell [15], each view should be sufficient to build a model and features belonging to different views should be as independent as possible. Note that even a single feature is sufficient to build a predictive model, since it is in fact the output score of a base method. We build the views by partitioning the feature set into subsets, such that correlated features fall in the same view and uncorrelated features fall in different views. In this way, each classification model focuses on the slight differences among correlated features, while the combination of the models built from different views captures more global underlying patterns.

Concerning *ii)*, the multi-view learning framework is able to exploit unlabeled examples to boost classification iteratively. This is especially important when few labeled examples are available. Moreover, the classifier we consider is able to build a classification model from only positive examples and from unlabeled examples, thus solving the problem of unavailability of negative examples.

Concerning *iii)*, the imbalance between labeled and unlabeled examples is attacked by resorting to an ensemble-based approach: the same approach is used to build a different model for each different view. This means that, for each view, instead of learning a single classifier, we learn an ensemble of classifiers.

Tackling the issues *i)-iii)* resolves many problematic aspects of the network reconstruction task, including the selection of a single network reconstruction method from the plethora of existing ones, as well as dealing with the small quantity and the low quality of (experimentally verified) data.

The method we propose, as we describe in detail in Section *Results and Discussion*, has been evaluated on synthetically generated data and on the datasets used in the Dream5 challenge [12]. In the first case, we combine the output of several methods which are based on the *relevance networks* approach [16]. This approach consists of two main steps: the construction of a similarity (or distance) matrix for the set of considered genes (defined according to some similarity/distance measure) and application of a *scoring scheme* over the constructed matrix, i.e., a function which “corrects” the similarity/distance scores, according to marginal probabilities and joint probabilities computed over the different genes. Moreover, we apply the time shifting technique [17] in order to infer the direction of the regulations from the similarity matrix. The measures considered for the construction of the similarity (or distance) matrix can be categorized into three different groups, based on the nature of the input data structure: measures on vectors, random variables, and symbolic dynamics. The Dream5 datasets have been addressed by a more heterogeneous set of network inference methods, which are based on regression, mutual information, correlation, Bayesian networks, meta/combined approaches and other (hybrid) approaches (see S1 Text for details).

## Methods

The problem of network reconstruction (also called link prediction in machine learning) is a long-standing challenge in modern information science, and many approaches to address it have been proposed in the machine learning literature in recent years. They can be based on the following different methods: relevance networks, clustering, Bayesian Networks, differential equations, Markov chains, probabilistic models, random walk processes and maximum likelihood. Details about these approaches are given by Lu and Zhou [18].

Although widely used, the above methods do not simultaneously consider most of the aspects that have been recognized as important in the study of complex networks. In particular, they do not simultaneously exploit the properties of the nodes, the properties of the links and the topological characteristics of the networks, such as the possible hierarchical organization [19], the community structure [20] and the homophily principle [21]. In this paper, we follow the basic idea of methods which combine the outcomes of more than one method, i.e., ensemble/community approaches [12]. In particular, we propose to *learn to combine* predictions provided by different link prediction algorithms that possibly (and individually) exploit different aspects, so that they are simultaneously considered. The framework we resort to is that of *stacked generalization* [14], where the outputs of different prediction algorithms are considered as features for a further run of a learning algorithm.

To the best of our knowledge, there are few papers [22, 23] that address the problem of learning to combine the predictions of links for network reconstruction by means of the stacked generalization approach. Whalen and Pandey [22] suggest to cluster classifiers with similar predictions and then learn a meta-classifier for each cluster, which predicts the score associated to each link. Final scores are obtained as the average of the scores returned by each meta-classifier. The main difficulty in the application of this approach in our context is that it does not consider the problems of learning from positive examples only and the imbalance of known/unknown labels (see issues *ii)* and *iii)* in Section *Introduction*). Pio et al [23] suggest to use semi-supervised learning to predict links in a bipartite graph. However, this work does not exploit the concept of multi-view learning for boosting the predictions for unknown cases and, thus, it does not distinguish among the different contributions of similar base methods (see issue *i)* in the *Introduction*).

With respect to Pio et al. [23], our work has some additional differences. First, we apply an iterative solution (similar to that adopted in co-training) that is able to exploit unlabeled examples to boost classification. This is in-line with the semi-supervised learning framework and is especially important when few labeled examples are available. Second, Pio et al. [23] aim at discovering microRNA-mRNA interaction networks and not gene regulatory networks. In this case, some (or few) positive examples of links are available. 3) While Pio et al. [23], extract links on bi-partite undirected graphs and require as input positive examples of interactions, the method proposed here operates on simple directed graphs and can work without positive example of links.

In this paper, we take inspiration from these two papers in order to deal with issues *i)-iii)*, arising for the specific task at hand. This section describes in detail the proposed method.

### Problem definition

Before describing our approach for learning to combine the predictions of several algorithms for network reconstruction, we introduce some notions and formally define the task we intend to solve. Let:

- *E* be the set of genes, which represent the nodes of the network;
- *x* = 〈*e*′, *e*′′〉 ∈ (*E* × *E*) be a (possible) interaction between the genes *e*′ and *e*′′, which represents a link in the network;
- *p*_*k*_(*x*) be the prediction score for the interaction *x*, returned by the *k*-th base method, 1 ≤ *k* ≤ *s*;
- *p*(*x*) = [*p*_1_(*x*), *p*_2_(*x*), …, *p*_*s*_(*x*)] be the vector of prediction scores associated with the interaction *x*;
- *l*(*x*) returns 1 if the existence of the interaction *x* is known and 0 if it is unknown (regardless of whether *x* exists or not);
- *L* = {*x* ∈ (*E* × *E*)|*l*(*x*) = 1} be the initial set of labeled interactions;
- *U* = (*E* × *E*)\*L* be the set of (initially) unlabeled interactions;
- *f*(*x*) be an ideal (target) function which returns 1 if *x* is an existing interaction (either known or unknown), and 0 otherwise.

The task we intend to solve is then defined as follows:

*Given:* a set of training examples {〈*p*(*x*), *l*(*x*)〉}_*x*_. *Find:* a function $f^{\prime }:R^{s}\to R$ which takes as input a vector of prediction scores *p*(*x*) and returns the probability that the interaction *x* exists. We have that $f^{\prime }\left(p\left(x\right)\right)\approx P\left(f\left(x\right)=1\right)$ or, in other terms, *f*′() approximates the probability distribution over the values of the ideal function *f*().

A direct consequence of the definition of the functions *f*(*x*) and *l*(*x*) is:

$$\begin{array}{c} P(f(x)=1|l(x)=1)=1 \end{array}$$ (1)

that is, only known existing interactions are labeled.

Our approach, called GENERE (which stands for GEne NEtwork REconstruction) works in three phases. In the first phase, we use the above definition to assign labels to examples in the first phase of our approach. In this phase, we also identify multiple views. As stated in the *Introduction*, we exploit a solution which is mainly based on the multi-view learning framework. In particular, we construct *V* views by partitioning the set of *s* base methods on the basis of their predicted scores, i.e., the values in {*p*(*x*)}_*x*_. The goal is to have the most similar base methods grouped in the same subset and the most dissimilar base methods in different subsets. In this way, the *v*-th view represents a new training set with the prediction scores obtained by the base methods falling into the *v*-th subset.

Once the views are built, the algorithm iteratively learns a classification model from each view (phase 2). The reliable predictions obtained by each classification model in one iteration are used, in the next iteration, to extend or correct the training set of the other views. The reliability of each prediction is estimated according to the average scores obtained over the different views (see Section *Phase 3—Combining the output of views* for details). The iterative process stops when the maximum number of iterations, *max*_*iter*, is reached. The final output scores (phase 3) are obtained by averaging the scores obtained by the *V* classifiers at the last iteration.

**Fig 1:**
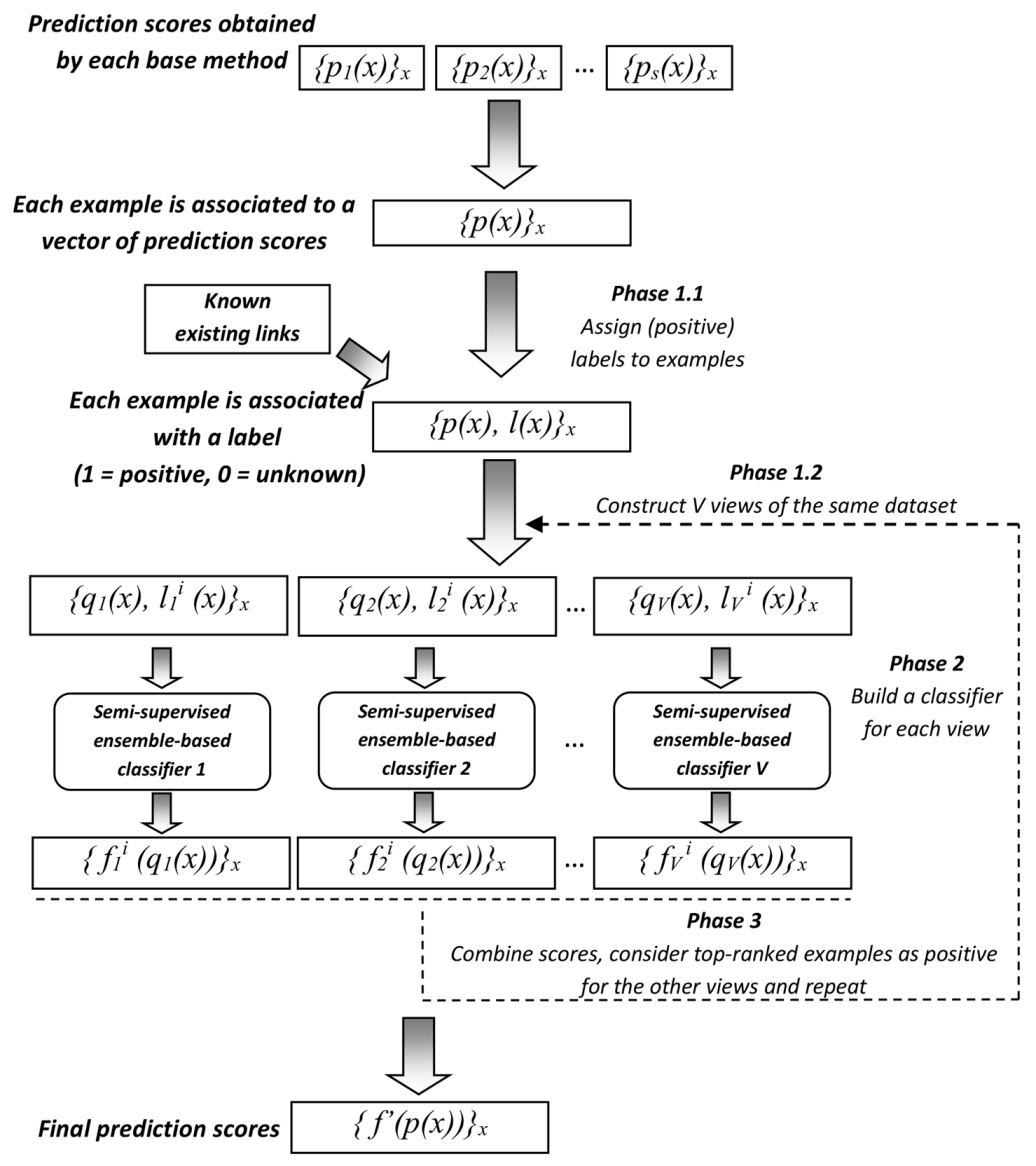
General workflow of GENERE approach that learns to combine the predictions of different GRN reconstruction methods.

Fig 1 shows a high-level description of the GENERE workflow, where *q*_*v*_(*x*) represents the vector of prediction scores obtained by the base methods in the *v*-th subset. Each of these phases is described in the following subsections.

### Phase 1—Assigning labels and identifying multiple views

Once the vector of prediction scores *p*(*x*) is initialized and *l*(*x*) is defined for each interaction *x*, the algorithm constructs *V* different views of the same dataset. Each view consists of the whole set of examples and one subset of the original set of features, where each feature represents the prediction score returned by one base method.

According to the original co-training setting [15] and works on multi-view learning [24], such views should be as independent as possible. For this reason, we apply a clustering algorithm to the features in order to obtain a partitioning which minimizes the inter-cluster similarity and, thus, the correlation between features belonging to different views. Although almost all clustering algorithms can, in principle, be applied to this task, we use two well-know algorithms: Principal Component Analysis (PCA) and K-means. While the first approach is tailored to partition features (and not examples), the second was appropriately modified in order to partition features. In PCA, after *V* principal components are identified, each feature is associated with the component with the highest contribution in the linear combination. In K-means, which is a centroid-based clustering algorithm, examples (features, in our case) are iteratively assigned to the most similar centroid and centroids are recomputed on the basis of the last assignments. Each feature is associated to a cluster based on the assignment in the last iteration of the algorithm.

In both cases, each feature is associated with one of the *V* clusters. Formally, given the vectors of prediction scores {*p*(*x*)}_*x*_, we apply PCA or K-means to identify a partition of features into *V* subsets. Each example is then represented by *V* (disjoint) vectors of prediction scores *q*_1_(*x*), *q*_2_(*x*), …, *q*_*V*_(*x*), each of which corresponds to a subset of features and contributes to build a different view (see Fig 1).

At the beginning of the process, each example of interaction *x* in the *v*-th view is represented by the vector of prediction scores *q*_*v*_(*x*) and is associated with the label *l*(*x*). During the process shown in Fig 1, the set of labeled interactions is iteratively extended with new examples considered as positive. If *L* = {*x* ∈ (*E* × *E*)|*l*(*x*) = 1} is the initial set of labeled interactions, we indicate as $L_{v}^{i}⊇L$ the set of labeled interactions for the *v*-th view, at the *i*-th iteration. More formally, $L_{v}^{i}=\left\{x\in \left(E\times E\right)|l_{v}^{i}\left(x\right)=1\right\}$, where $l_{v}^{i}\left(x\right)=1$ if the interaction *x* is labeled at the iteration *i* for the view *v*, 0 otherwise. Note that each $L_{v}^{i}$ is always a super-set of (or equal to) *L*: although decisions taken at an iteration can be *retracted* in the subsequent iterations, examples considered as positive in the first iteration, i.e., belonging to *L*, are always considered as positive examples (see Section *Phase 3—Combining the output of views* below for details). Accordingly, we indicate as $U_{v}^{i}\subseteq U$ the set of unlabeled interactions at the iteration *i* for the view *v*. Formally, $U_{v}^{i}=\left(E\times E\right)\setminus L_{v}^{i}$.

At the first iteration, $L=L_{1}^{0}=\ldots =L_{V}^{0}$ and $U=U_{1}^{0}=\ldots =U_{V}^{0}$. For the subsequent iterations (*i* > 0), both $L_{v}^{i}$ and $U_{v}^{i}$ can contain different examples, since they are iteratively updated according to the output obtained from the other views (see Section *Phase 3—Combining the output of views*).

### Phase 2—Building a classifier for each view

As it can be observed in Fig 1, we build a different classifier from each view, by exploiting information conveyed by both known existing interactions (i.e., positively labeled examples) and unknown interactions (i.e., unlabeled examples).

The goal of the *v*-th classifier at the *i*-th iteration is to identify a function $f_{v}^{i}\left(q_{v}\left(x\right)\right)$ which approximates the probability that *f*(*x*) = 1, that is, $f_{v}^{i}\left(q_{v}\left(x\right)\right)\approx P\left(f\left(x\right)=1\right)$. As suggested by Elkan and Noto [25], $f_{v}^{i}\left(q_{v}\left(x\right)\right)$ can be identified by exploiting Eq (1) and the Bayes’ rule as follows:

$$\begin{array}{ccc} P(l_{v}^{i}\left(x\right)=1) & = & P(f\left(x\right)=1\wedge l_{v}^{i}\left(x\right)=1)=\\ & = & P(l_{v}^{i}\left(x\right)=1|f\left(x\right)=1)\cdot P\left(f\left(x\right)=1\right) \end{array}$$ (2)

Therefore:

$$\begin{array}{c} f_{v}^{i}\left(q_{v}\left(x\right)\right)\approx P\left(f\left(x\right)=1\right)=\frac{P(l_{v}^{i}\left(x\right)=1)}{P(l_{v}^{i}\left(x\right)=1|f\left(x\right)=1)} \end{array}$$ (3)

At the *i*-th iteration, we can assume that examples for which we know the label, provided by the *v*-th classifier, are correctly classified as positive. This assumption can be made since at the next iteration we can retract decisions (as discussed in Section *Phase 3—Combining the output of views*).

In Eq (3), both the numerator and the denominator can be estimated by means of a so-called “non-traditional” classifier, whose definition is explained in the following.

#### Estimating $P(l_{v}^{i}\left(x\right)=1)$ and $P(l_{v}^{i}\left(x\right)=1|f\left(x\right)=1)$

In order to estimate $P(l_{v}^{i}\left(x\right)=1)$ and $P(l_{v}^{i}\left(x\right)=1|f\left(x\right)=1)$, we build a so-called “non-traditional” classifier for each view *v* at the iteration *i*, whose output is the probability that an example is labeled, i.e., $P(l_{v}^{i}\left(x\right)=1)$. Note that any probabilistic classifier can be adopted for this task. In this work, we adopt an SVM-based classifier, i.e., LibLinear [26] with Platt Scaling, mainly for the following reasons: *1)* it has a (relatively) good computational efficiency, especially in the prediction phase, which is based on a very limited number of examples (support vectors); *2)* it is robust to noise and to feature redundancy [27]; *3)* its effectiveness (with Platt scaling) has already been proved in the semi-supervised setting [25]. However, every other algorithm that exhibits similar properties can be plugged into our framework.

Formally, the task solved by each non-traditional classifier *v* at iteration *i* is defined as follows: Given the set of training examples ${\left\{\langle q_{v}\left(x\right),l_{v}^{i}\left(x\right)\rangle \right\}}_{x}$, the task is to find a probability function $g_{v}^{i}\left(q_{v}\left(x\right)\right)$ which returns the probability that the interaction *x* is labeled, i.e. $g_{v}^{i}\left(q_{v}\left(x\right)\right)\approx P\left(l_{v}^{i}\left(x\right)=1\right)$. This means that $g_{v}^{i}\left(q_{v}\left(x\right)\right)$ can be used to estimate both the numerator and the denominator of Eq (3) as follows:

$$\begin{array}{c} f_{v}^{i}\left(q_{i}\left(x\right)\right)\approx \frac{g_{v}^{i}\left(q_{v}\left(x\right)\right)}{\frac{1}{\left|L_{v}^{i}\right|}\sum_{x^{\prime }\in L_{v}^{i}}g_{v}^{i}\left(q_{v}\left(x^{\prime }\right)\right)} \end{array}$$ (4)

In the denominator of Eq (4), we assume that all the labeled examples are taken completely randomly from all the positive examples. Formally:

$$\begin{array}{c} P(l_{v}^{i}\left(x\right)=1|f\left(x\right)=1)=P(l_{v}^{i}\left(\cdot \right)=1|f\left(\cdot \right)=1) \end{array}$$ (5)

In other words, $P(l_{v}^{i}\left(x\right)=1|f\left(x\right)=1)$ is independent of the specific interaction *x*. Hereafter, we adopt the notation P(l(·)=1|f(·)=1) to represent the fact that the probability of a positive interaction is labeled is independent of the specific interaction *x*. This assumption is in line with typical assumptions made by methods that learn from only positive examples [25] and allows us to exploit $g_{v}^{i}\left(q_{v}\left(x\right)\right)$ also for the computation of the denominator of Eq (4). In particular, we estimate $P(l_{v}^{i}\left(x\right)=1|f\left(x\right)=1)$ as the average of $g_{v}^{i}\left(q_{i}\left(x\right)\right)$ over all the positive examples at the *i*-th iteration and at the *v*-th view:

$$\begin{array}{c} P\left(l_{v}^{i}\left(x\right)=1|f\left(x\right)=1\right)=P\left(l_{v}^{i}\left(\cdot \right)=1|f\left(\cdot \right)=1\right)\approx \frac{\sum_{x^{\prime }\in L_{v}^{i}}g_{v}^{i}\left(q_{v}\left(x^{\prime }\right)\right)}{\left|L_{v}^{i}\right|} \end{array}$$ (6)

Differently from Elkan and Noto [25], we also have to deal with the problem of imbalanced class distributions. This issue is considered in the next subsection.

#### Ensemble Learning of $g_{v}^{i}\left(\cdot \right)$

In this subsection, we describe the approach we adopt to deal with the imbalance between the number of labeled and unlabeled examples. This approach, following Pio et al. [23], exploits a sampling procedure which is similar to that used in bootstrap estimation [28] and bagging [29].

For each view *v* at a given iteration *i*, we learn a set of *W* non-traditional classifiers ${\left\{g_{vj}^{i}\left(q_{v}\left(x\right)\right)\right\}}_{j=1,2,\ldots ,W}$ whose outputs are combined to obtain $g_{v}^{i}\left(q_{v}\left(x\right)\right)$. Each classifier is built from the set of examples $L_{v}^{i}\cup U_{vj}^{i}$, that is, from all the labeled examples $L_{v}^{i}$ and from the set $U_{vj}^{i}$, with *j* = 1, 2, …, *W*. $U_{vj}^{i}$ is a subset of the unlabeled examples $U_{v}^{i}$, obtained by a random sampling, with replacement, of *n* examples from $U_{v}^{i}$. The proportion of unlabeled examples in each $U_{vj}^{i}$ is $\frac{n}{|U_{v}^{i}|}$, where *n* is a user-defined parameter. The *W* samples ${\left\{U_{vj}^{i}\right\}}_{j}$ are neither mutually exclusive nor exhaustive, i.e., they do not partition the set $U_{v}^{i}$. Differently from data partitioning, which is affected by only one parameter *W* (the number of subsets), the data sampling procedure used in this work is controlled by two parameters: *n* and *γ*. The first parameter represents the number of unlabeled examples in each sample and can be chosen reasonably on the basis of the number of labeled examples, so that the imbalance is mitigated. The second parameter represents the percentage of unlabeled examples we intend to take into account (i.e., to cover) and influences the number of samples *W* (i.e., the higher the percentage of examples to cover, the higher the number of samples *W*). This parameter is necessary since the sampling with replacement would require an infinite number of samples to guarantee that each example is selected at least once.

Once the *W* classifiers are learned, each function $g_{vj}^{i}\left(q_{v}\left(x\right)\right)$ is applied to obtain an estimate of $P(l_{v}^{i}\left(x\right)=1)$ for all the examples in $U_{vj}^{i}$. Since the same unlabeled example can belong to more than one sample, we average the outputs of the classifiers as follows:

$$\begin{array}{c} g_{v}^{i}\left(q_{v}\left(x\right)\right)=\underset{\left\{j\;|\;U_{vj}^{i}\;\text{contains}\;x\right\}}{average}g_{vj}^{i}\left(q_{v}\left(x\right)\right) \end{array}$$ (7)

While ensemble learning approaches have been already used for GNR and, in particular, for stability selection [30], there is a clear difference between these approaches and GENERE, both in terms of algorithm and goals. In terms of algorithm, our method clusters features and builds an ensemble for each cluster. Differently, stability selection runs feature selection algorithms many times, resampling examples and variables at each run, and computes the frequency with which each variable is selected across the runs. In terms of goals of the use of ensemble techniques, GENERE uses them to overcome the imbalance between positive and unlabelled examples and exploit separately the different information coming from different features. In contrast, stability selection aims to identify the best features.

### Phase 3—Combining the output of views

Once all the functions $f_{v}^{i}\left(q_{v}\left(x\right)\right)$ for the iteration *i* are computed (see Eq (4)), we combine their outputs in order to update the set of (positively) labeled examples for the next iteration (i.e., the (*i* + 1)-th iteration). In the original co-training framework, the best examples to be chosen as training examples for the next iteration are those with the highest reliability in the classification. In our case, since we only learn from positive examples of interactions, we simply choose those for which we have, on average over the different views, the highest scores. In particular, to update the set of labeled examples of the view *v* at the iteration *i* + 1, we average the outputs $f_{z}^{i}\left(q_{z}\left(x\right)\right)$ over all the views *z* ≠ *v*, for each interaction *x*:

$$\begin{array}{c} f_{comb_{v}}^{i}\left(x\right)=\frac{1}{W-1}\sum_{z=1,2,\ldots W;\;z\neq v}f_{z}^{i}\left(q_{z}\left(x\right)\right) \end{array}$$ (8)

Formally, the set of labeled examples of the view *v* at the iteration *i* + 1, i.e. $L_{v}^{i+1}$, is built by taking a given percentage *δ* of the top-ranked examples according to $f_{comb_{v}}^{i}\left(x\right)$. Note that examples belonging to $L_{v}^{i}$ do not necessarily belong to $L_{v}^{i+1}$. This means that decisions taken at an iteration can be *retracted* in subsequent iterations. However, examples considered as positive in the first iteration, i.e., belonging to *L*, are always considered as positive examples, since we assume that they are experimentally verified.

### Additional Remarks

Note that the algorithm works on samples of links (not nodes) and reconstructs the network at each iteration. This implies that the algorithm is able to work with different types of underlying networks. In particular, if the underlying type of the network is scale-free (as several authors claim is the case of eukaryotic and prokaryotic organisms [31–33]), the algorithm starts from hubs (since they have higher probability to be selected during sampling) and considers additional nodes during the next iterations. If the underlying type of the network is based on the Erdös-Rényi random graph model, the algorithm would, at the first iteration, identify subnetworks on the basis of the generated samples. In this case, if all the links have the same score, each link (and each node) has the same probability to be selected. Starting from the ones selected in the first iteration, the algorithm considers additional candidate links at subsequent iterations, on the basis of the new samples generated.

## Results and Discussion

In this section, we evaluate the effectiveness of our approach which has been implemented in the system GENERE (GEne NEtwork REconstruction). With this aim, we perform gene network inference and compare the obtained gene networks to a given gold standard.

The main goal of our evaluation is to prove the effectiveness of the proposed approach. We compare it with the following state-of-the-art combination strategies:

- **Borda**, proposed by Marbach et al. in [12], which averages the ranks of the outputs of the considered base methods.
- **TopKNet (TopK)**, which takes, for each interaction, the *k*-th highest rank among the different base methods [13]. In this evaluation, we only consider the results obtained by varying *k* in the interval [1, 20]. In fact, according to results reported by Hase et al. [13], the best results are obtained in this interval and there is no deterioration before *k* = 20, whereas there is a clear deterioration for *k* > 20.

Moreover, in order to assess the effectiveness of the iterative multi-view learning framework, we also report a comparison with an adapted version of the algorithm proposed by Pio et al. [23] where, instead of generating bipartite undirected graphs, we generate directed graphs. This algorithm is actually a simplified version of GENERE, where one single view is built (i.e., *V* = 1) and one single iteration is performed (i.e., *max*_*iter* = 1). We denote this algorithm **1VI** (One View, One Iteration).

### Data description and generation

Below we describe in details the datasets and briefly mention the base methods. The evaluation of our approach has been performed on *i)* the collection of synthetic datasets used by Hempel et al. [17], which are generated with the tool SynTReN (Synthetic Transcriptional Regulatory Networks) [34], and on *ii)* the datasets used in the DREAM5 challenge [12]. More details on the base methods are given in S1 Text.

#### The SynTReN datasets

were obtained from gold-standard gene networks, where genes are represented with their expression data. The datasets were generated by the tool SynTReN on the basis of the well-defined regulatory networks of the organisms *E. coli* and *S. cerevisiae* (henceforth *Yeast*) [17]. In particular, SynTReN: *1)* selects connected sub-networks of the input networks and *2)* generates gene expression data which best fit the underlying network structure. While interactions of the selected sub-networks are considered as gold standard for our evaluation, the generated expression data are used as input features for the considered base methods and, thus, are indirectly exploited by our approach. Different base methods have been applied to the SynTReN and DREAM5 datasets.

We consider sub-networks of 100, 150 and 200 genes, characterized by 121, 202 and 303 existing links with an average node degree of 2.42, 2.46 and 3.03, respectively. This is necessary in order to evaluate the sensitivity of the algorithms to the size of the networks. Note that, in order to guarantee consistency between sub-networks and expression data, SynTReN generates different expression data for each selected sub-network.

In the generation of expression data, SynTReN exploits Michaelis-Menten and Hill kinetics so that the generated expression data are very similar to real microarray mRNA measurements [34]. For both *E. coli* and *Yeast*, the expression data are represented by 10 biological conditions. In order to evaluate the robustness to noise, we exploit SynTReN’s capability to introduce noise in expression data. Such noise is additive, lognormally-distributed and consists of stochastic variations that are unrelated to the applied experimental procedures. In our experiments, we considered three levels of noise: 0.0 (deterministic—without noise), 0.1 and 0.5. These values represent the *σ* parameter of the lognormal distribution ∼logX(0,σ) according to which the noise is generated. For each configuration, 6 technical replicates have been generated and the expression data associated with each gene is obtained as the average over the replicates. This is necessary to cope with the non-deterministic nature of the SynTReN data generation algorithm.

The considered base methods follow the relevance network approach [16], which, as stated in the introduction, consists in the construction of a similarity matrix and in the application of a scoring scheme. A detailed description of each similarity/distance measure and scoring scheme, as well as the advantages and disadvantages of all the combinations of similarity/distance measures and scoring schemes can be found in S1 Text (cf. also Hempel et al. [17]). In our experiments, all the possible combinations of all the 23 measures and 6 scoring schemes mentioned in S1 Text are considered, resulting in 138 combinations (14 have been discarded since they returned no interactions—see S1 Fig), each of which is considered as a base method. This is different from Hempel et al. [17], where only 50 combinations are considered.

#### The DREAM5 datasets

were obtained from the DREAM5 challenge set of networks. In particular, we considered Network1, Network3 and Network4 (Network2 was not considered for the computation of scores during the challenge). Network1 is a synthetic dataset (*D5InSilico* in our experiment). Network3 and Network4 are based on Affymetrix gene expression data of the organisms *E.coli* (*D5EColi* in our experiment) and *S. cerevisiae* (*D5Yeast* in our experiment), taken from the Gene Expression Omnibus (GEO) database [35], collected under a wide range of biological conditions.

The microarrays have been uniformly normalized using Robust Multichip Averaging (RMA) [36]. These data have been processed by the DREAM5 participants’ methods in order to generate scores. The methods are grouped in six groups, based on the principle and methodology of scores derivation [12]. Further details can be found in S1 Table. From the DREAM5 challenge, we also obtained the networks that we use as gold standard (only for evaluation purposes). Further details are given by Marbach et al. [12].

These datesets are larger than the SynTReN datasets. In particular, *D5InSilico* contains 1643 genes, *D5EColi* contains 4511 genes and *D5Yeast* contains 5950 genes. All these datasets are processed by considering the scores obtained by the 35 base methods used in the competition (see S1 Text).

### Experimental setting

In the experiments, the value of *γ* has been chosen in order to cover the maximum number of unlabeled examples, without resulting in an extremely high number of samples. Pio et al. [23] investigated the effect of *γ*. Although the goal of that study is different and the method is non-iterative and not based on the multi-view learning approach, the results concerning the performance for different values of *γ* are still applicable to our case. Since the best results are obtained with the highest possible value of *γ*, we set *γ* = 0.99. The value of *max*_*iter* has been set to a relatively high value (30 for the SynTReN datasets and 5 for the DREAM5 datasets) in order to have enough information to empirically evaluate the best number of iterations.

As for *n* (the number of unlabeled examples sampled for each classifier), *δ* (percentage of top-ranked examples to be considered as positive examples in the next iteration) and *V* (number of views), we performed a preliminary experiment to evaluate their effect on the results.

We can observe that $n=|L_{v}^{i}|$ guarantees, for each view and for each iteration, a perfect balance between labeled and unlabeled examples. Setting $n>|L_{v}^{i}|*2$ can potentially lead to problems in the probability estimations. Moreover, considering more than 4-5 views generally leads to a decrease in accuracy, probably due to high fragmentation of the available features. Finally, considering *δ* > 1.0% generally leads to including noisy examples in the subsequent iterations. For these reasons, we performed the experiments with the following sets of values: $n\in \{|L_{v}^{i}|,|L_{v}^{i}|*2\}$, *δ* ∈ {0.5%, 1.0%} and *V* ∈ {1, 2, 3, 4, 5}.

In order to guarantee a fair comparison with Borda and TopK, which are unsupervised, we forced our system and 1VI to work in the worst case scenario, that is, in the scenario in which there is no confirmed interaction at the beginning of the learning process. In order to provide our algorithm some information to start with, at the very beginning we label as positive the first “best” interactions. In particular, *L* is initialized with the top *δ* examples, ranked according to their average score.

In our analysis, we performed experiments with both clustering algorithms, that is, PCA and K-means. The inferred gene networks are evaluated in terms of the Area Under the ROC Curve (AUROC) [37] and the Area Under the Precision-Recall Curve (AUPRC) [38], with respect to the gold standard. These measures allow us to evaluate the predictions of the studied approaches independently of the threshold (on the output score) used to consider an interaction as positive.

### Results

In sum, we use 21 datasets for network reconstruction. Namely, for each of the two organisms (E. coli, Yeast), there are 9 SynTReN datasets (3 sizes: 100, 150 and 200 genes, 3 noise levels: 0, 0.1, and 0.5), making for 18 datasets. In addition, we have the three DREAM5 datasets.

For each of the SynTReN datasets, we first run the 138 base (relevance network) methods to obtain the appropriate scores. For the DREAM5 datasets, we use the scores produced by the 35 base methods as provided by the competition organizers. The dataset and base method details are provided in S1 Text.

**Fig 2:**
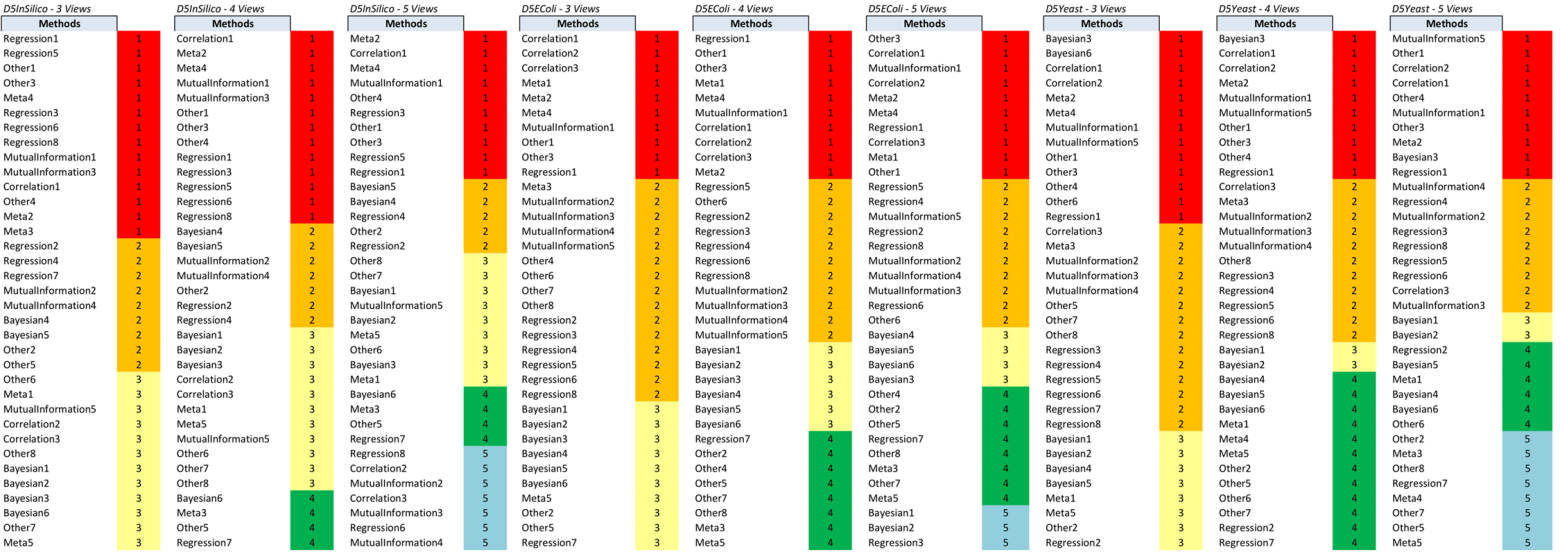
The views obtained on the DREAM5 dataset by clustering using PCA.

**Fig 3:**
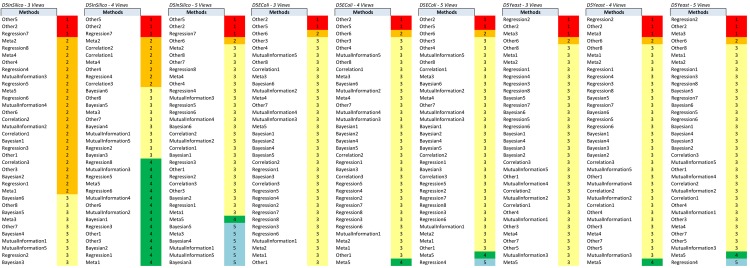
The views obtained on the DREAM5 dataset by clustering using K-means.

Starting from the scores produced by the base methods, our method GENERE performs clustering of the base methods into different numbers of clusters (1, 2, 3, 4, 5) to identify the appropriate views (groups of base methods), for each dataset separately. The PCA and K-means clustering algorithms are used for this purpose. The identified views for the DREAM5 datasets are given in Figs 2 and 3, and for a representative sample of SynTReN datasets (without noise) in the figures in S1 Fig.

After the appropriate clusters (sets of views) have been identified, the iterative semi-supervised part of the GENERE approach is applied, producing combined scores at each iteration. GENERE is run for up to 30 iterations for the SynTReN datasets and for up to 5 iterations for the DREAM5 datasets. AUROC and AUPRC values were calculated from the combined scores produced at each iteration (0-30 and 0-5, respectively) and are given in S1 Table. Note that a separate run is performed for each clustering (identified by PCA or K-means, containing 1, 2, 3, 4, or 5 views/clusters), i.e., for a total of 10 clusterings for each dataset.

Two different values of the *δ* parameter are considered in GENERE, leading to 20 rows of results for each dataset in the table in S1 Table. Each row in the table corresponds to a combination of a dataset, a clustering and a *δ* value. The rows for the DREAM5 datasets have 6 columns of AUROC/AUPRC values for the different numbers of iterations (0-5) and the rows for the SynTReN datasets 31 columns (0 to 30 iterations).

The results in S1 Table are obtained by using the setting $n=|L_{v}^{i}|$. An additional analysis has been performed in order to understand the effect of *n*, where we also consider both $n=|L_{v}^{i}|$ and $n=2*|L_{v}^{i}|$. GENERE is run for 5 iterations and the results are given in the same format as in S1 Table. The results obtained for the two values of *n* are compared by using the Wilcoxon signed-rank test. In terms of AUPRC there is no clear difference between the two, while in terms of AUROC, the results with $n=|L_{v}^{i}|$ are better than results with $n=2*|L_{v}^{i}|$. This was somehow expected, since keeping the balance between labeled and unlabeled examples of links helps the algorithm to better discriminate between them.

**Fig 4:**
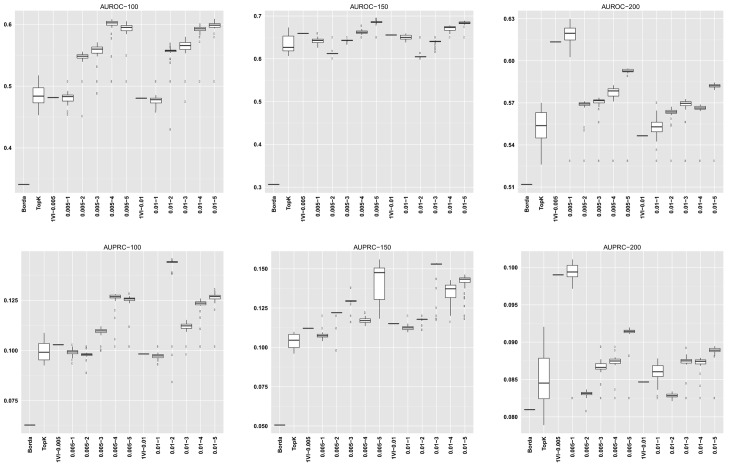
AUROC and AUPRC graphs for the SynTReN EColi dataset with noise level 0.0. The three columns of graphs represent results obtained with different numbers of genes in the dataset (100, 150 and 200, from left to right). Each graph represents box plots for (from left to right) Borda, TopK, 1VI with *δ* = 0.005, GENERE with *δ* = 0.005 and *V* ∈ {1, 2, 3, 4, 5}, 1VI with *δ* = 0.01, GENERE with *δ* = 0.01 and *V* ∈ {1, 2, 3, 4, 5}. The views are obtained with K-means. The boxplots for GENERE depict the distribution of AUROC/AUPRC values obtained by varying the number of iterations in [1, 30]. The boxplots for TopK depict the distribution of AUROC/AUPRC obtained by varying the value of k in [1, 20].

**Fig 5:**
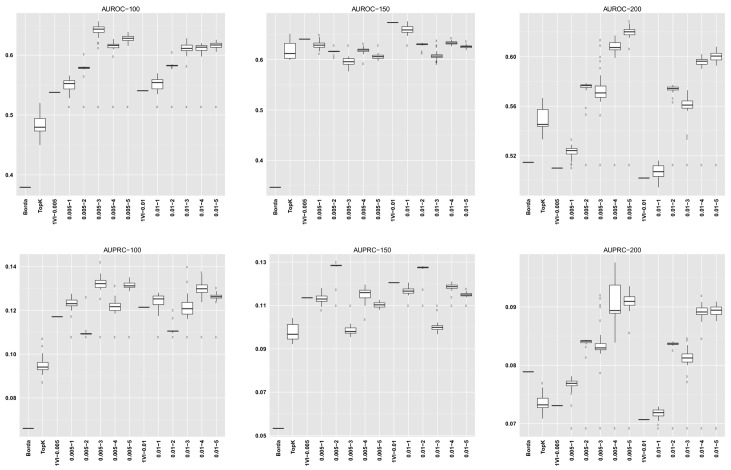
AUROC and AUPRC graphs for the SynTReN EColi dataset with noise level 0.1. The three columns of graphs represent results obtained with different numbers of genes in the dataset (100, 150 and 200, from left to right). Each graph represents box plots for (from left to right) Borda, TopK, 1VI with *δ* = 0.005, GENERE with *δ* = 0.005 and *V* ∈ {1, 2, 3, 4, 5}, 1VI with *δ* = 0.01, GENERE with *δ* = 0.01 and *V* ∈ {1, 2, 3, 4, 5}. The views are obtained with K-means. The boxplots for GENERE depict the distribution of AUROC/AUPRC values obtained by varying the number of iterations in [1, 30]. The boxplots for TopK depict the distribution of AUROC/AUPRC obtained by varying the value of k in [1, 20].

**Fig 6:**
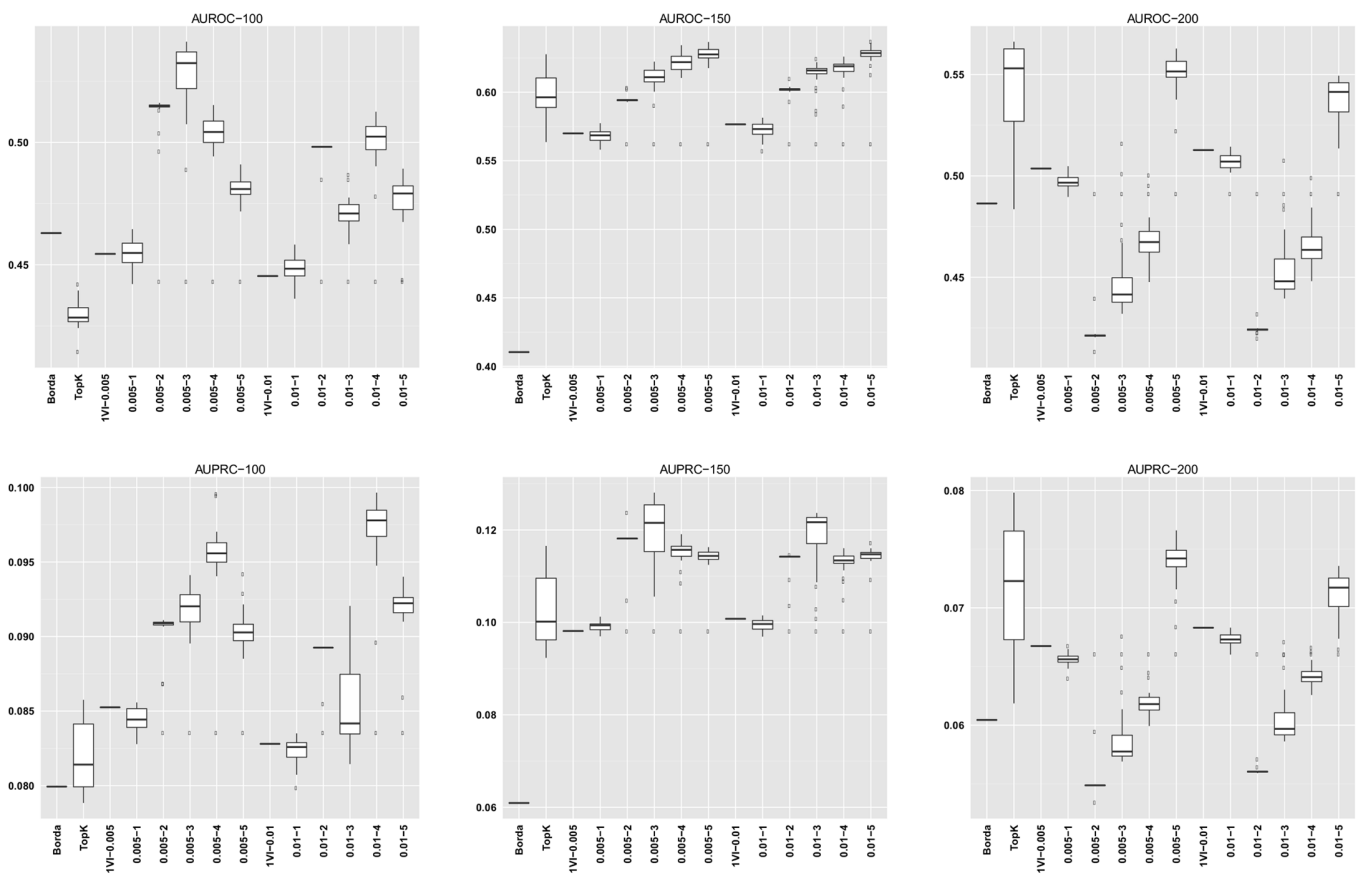
AUROC and AUPRC graphs for the SynTReN EColi dataset with noise level 0.5. The three columns of graphs represent results obtained with different numbers of genes in the dataset (100, 150 and 200, from left to right). Each graph represents box plots for (from left to right) Borda, TopK, 1VI with *δ* = 0.005, GENERE with *δ* = 0.005 and *V* ∈ {1, 2, 3, 4, 5}, 1VI with *δ* = 0.01, GENERE with *δ* = 0.01 and *V* ∈ {1, 2, 3, 4, 5}. The views are obtained with K-means. The boxplots for GENERE depict the distribution of AUROC/AUPRC values obtained by varying the number of iterations in [1, 30]. The boxplots for TopK depict the distribution of AUROC/AUPRC obtained by varying the value of k in [1, 20].

**Fig 7:**
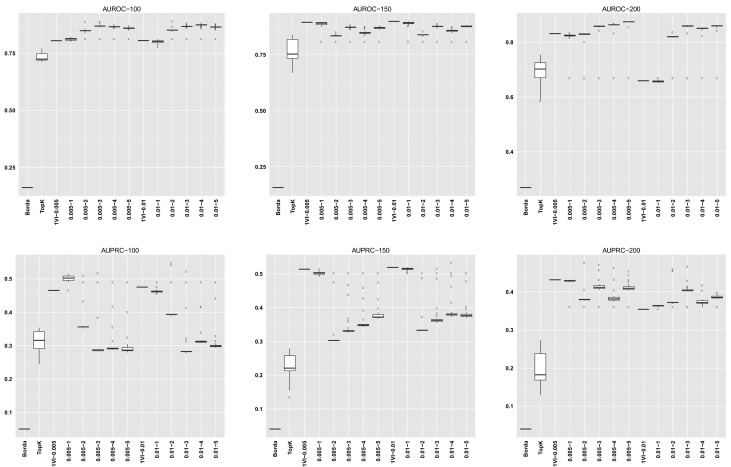
AUROC and AUPRC graphs for the SynTReN Yeast dataset with noise level 0.0. The three columns of graphs represent results obtained with different numbers of genes in the dataset (100, 150 and 200, from left to right). Each graph represents box plots for (from left to right) Borda, TopK, 1VI with *δ* = 0.005, GENERE with *δ* = 0.005 and *V* ∈ {1, 2, 3, 4, 5}, 1VI with *δ* = 0.01, GENERE with *δ* = 0.01 and *V* ∈ {1, 2, 3, 4, 5}. The views are obtained with K-means. The boxplots for GENERE depict the distribution of AUROC/AUPRC values obtained by varying the number of iterations in [1, 30]. The boxplots for TopK depict the distribution of AUROC/AUPRC obtained by varying the value of k in [1, 20].

**Fig 8:**
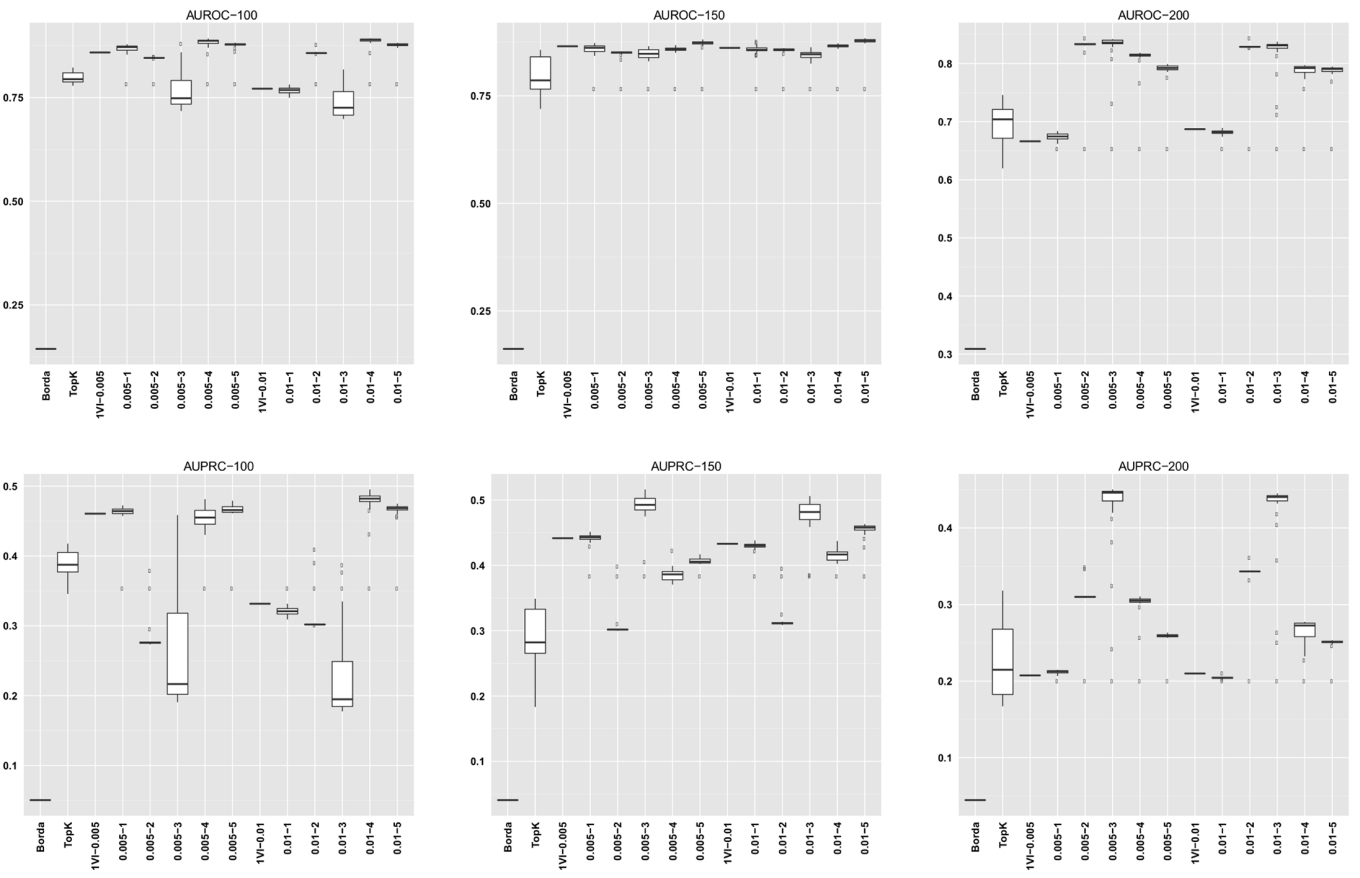
AUROC and AUPRC graphs for the SynTReN Yeast dataset with noise level 0.1. The three columns of graphs represent results obtained with different numbers of genes in the dataset (100, 150 and 200, from left to right). Each graph represents box plots for (from left to right) Borda, TopK, 1VI with *δ* = 0.005, GENERE with *δ* = 0.005 and *V* ∈ {1, 2, 3, 4, 5}, 1VI with *δ* = 0.01, GENERE with *δ* = 0.01 and *V* ∈ {1, 2, 3, 4, 5}. The views are obtained with K-means. The boxplots for GENERE depict the distribution of AUROC/AUPRC values obtained by varying the number of iterations in [1, 30]. The boxplots for TopK depict the distribution of AUROC/AUPRC obtained by varying the value of k in [1, 20].

**Fig 9:**
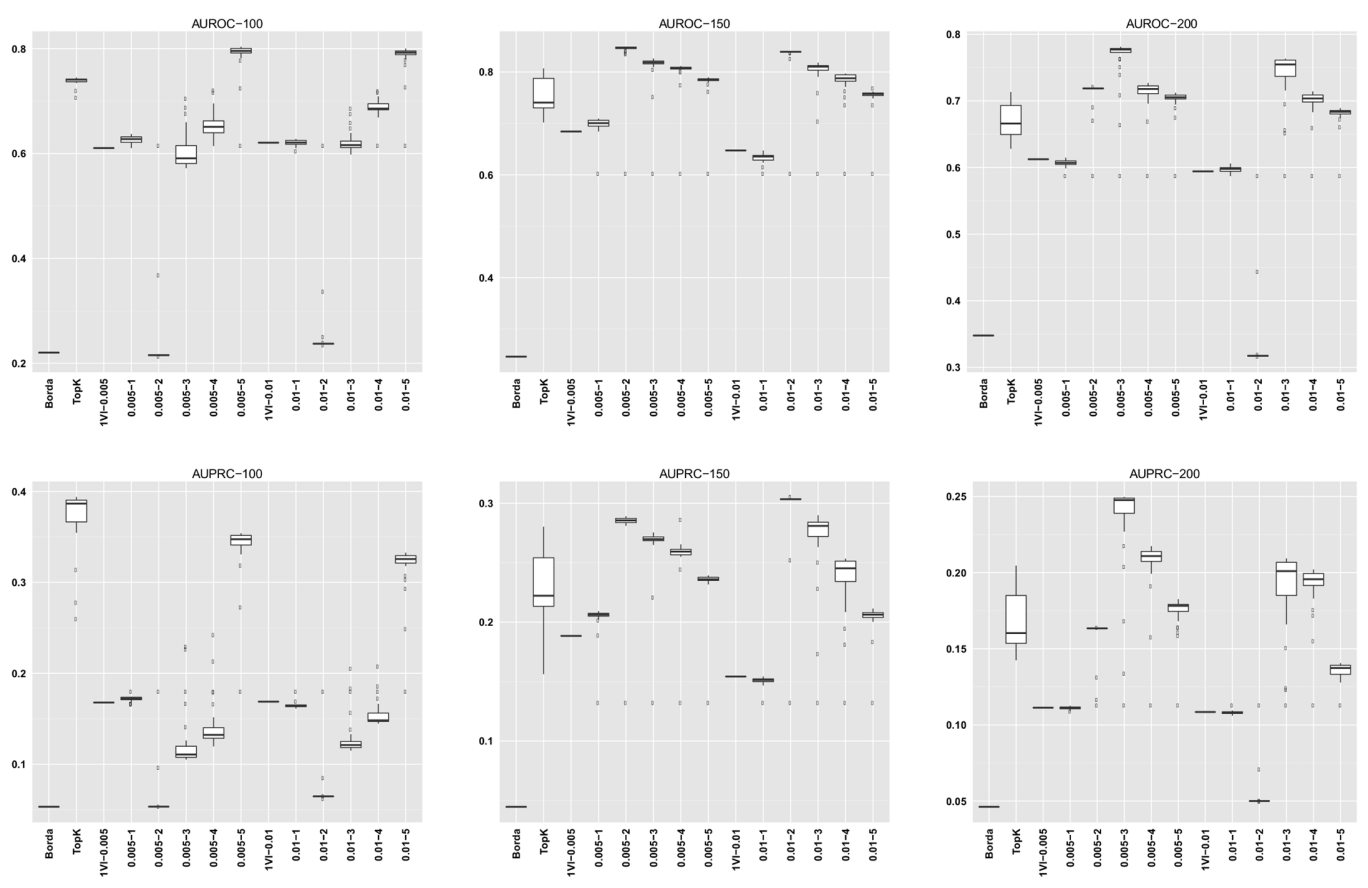
AUROC and AUPRC graphs for the SynTReN Yeast dataset with noise level 0.5. The three columns of graphs represent results obtained with different numbers of genes in the dataset (100, 150 and 200, from left to right). Each graph represents box plots for (from left to right) Borda, TopK, 1VI with *δ* = 0.005, GENERE with *δ* = 0.005 and *V* ∈ {1, 2, 3, 4, 5}, 1VI with *δ* = 0.01, GENERE with *δ* = 0.01 and *V* ∈ {1, 2, 3, 4, 5}. The views are obtained with PCA. The boxplots for GENERE depict the distribution of AUROC/AUPRC values obtained by varying the number of iterations in [1, 30]. The boxplots for TopK depict the distribution of AUROC/AUPRC obtained by varying the value of k in [1, 20].

**Fig 10:**
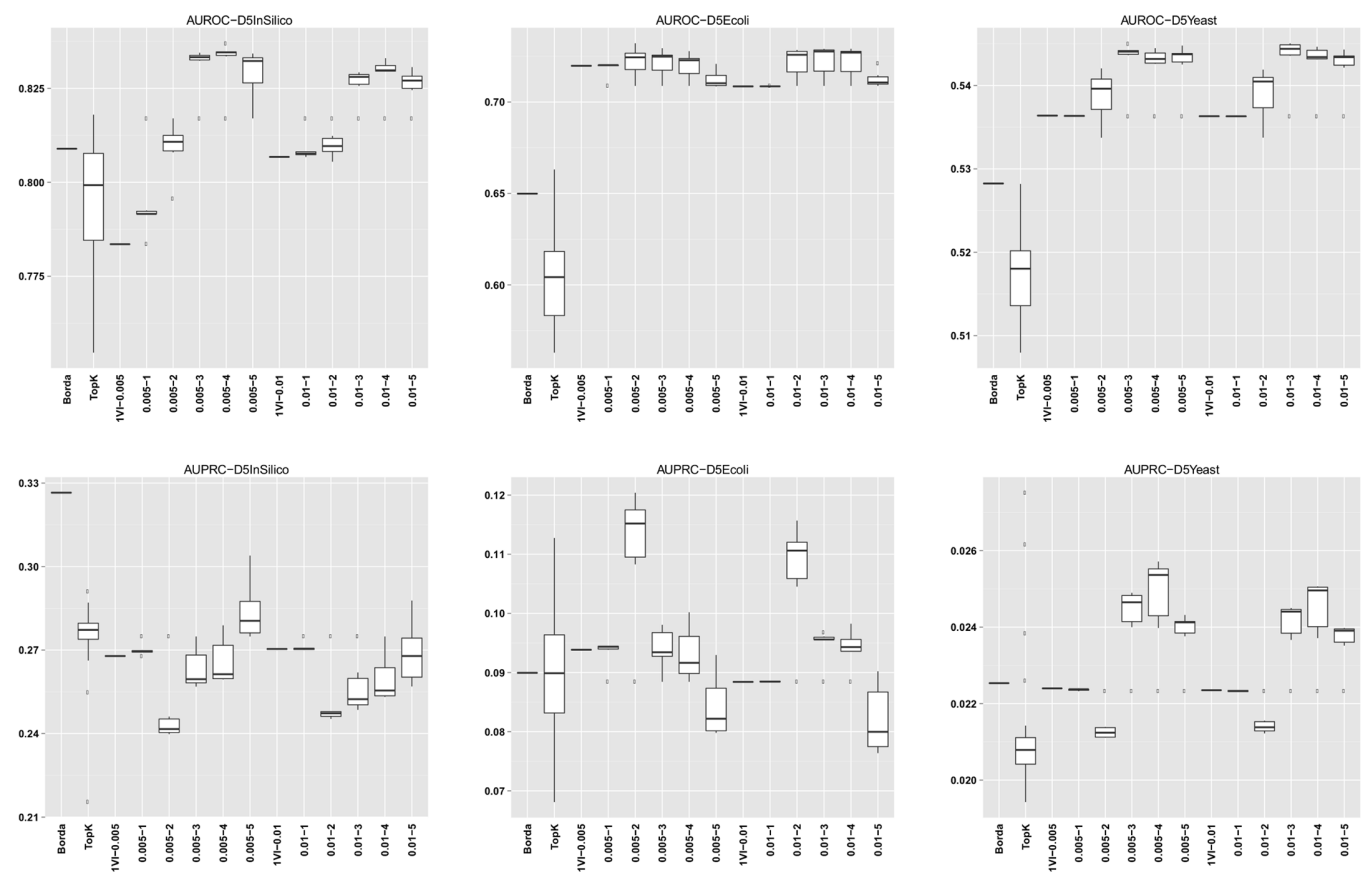
AUROC and AUPRC graphs for the DREAM5 datasets. The three columns of graphs represent results obtained on D5InSilico, D5EColi and D5Yeast, from left to right. Each graph represents box plots for (from left to right) Borda, TopK, 1VI with *δ* = 0.005, GENERE with *δ* = 0.005 and *V* ∈ {1, 2, 3, 4, 5}, 1VI with *δ* = 0.01, GENERE with *δ* = 0.01 and *V* ∈ {1, 2, 3, 4, 5}. The views are obtained with K-means. The boxplots for GENERE depict the distribution of AUROC/AUPRC values obtained by varying the number of iterations in [1, 5]. The boxplots for TopK depict the distribution of AUROC/AUPRC obtained by varying the value of k in [1, 20].

For GENERE, we summarize the distribution of AUROC/AUPRC values across the different numbers of iterations. The first five columns in each dataset row corresponding to GENERE results give the min, 1st quartile, median, 3rd quartile and the maximum AUROC/AUPRC values, respectively. These are succintly depicted via the boxplots in Figs 4–10 and the figures given in S2 Fig.

We compare the results of GENERE with those of three competing methods: Borda, TopK and 1VI. The latter is identical to GENERE with 1 view and one iteration and its results are not listed separately. To obtain the results of the other two competing methods, we first combine the scores provided by the base methods by using Borda, yielding one entry for each dataset given in one column. We then repeat the same exercises using TopK, varying K from 1 to 20 and record the results for each K in 20 different columns: These are also summarized via boxplots and the appropriate additional five columns are included in S1 Table.

The comparison between GENERE and its competitors is depicted in the boxplots in Figs 4–10 and the figures given in S2 Fig, as well as in Tables 1–6. Tables 1–4 give the results of the Wilcoxon signed rank test comparing the performance in terms of AUROC and AUPRC, respectively. Tables 5 and 6 give the average percentage of performance improvement of GENERE as compared to its competitors, for performance measured as AUROC and AUPRC, respectively.

**Table 1:** Adjusted *p*-values of the Wilcoxon signed rank test performed to compare GENERE with Borda, TopK and 1VI in terms of AUROC. Statistically significant values (<0.05) are shown in bold. Italic values indicates that the competitor outperforms GENERE. *p*-values are computed by considering the median value over the results obtained with different values of the number of iterations for GENERE, and over the results obtained with different values of k for TopK. Views are obtained with PCA.

GENERE vs	*δ*	1 View	2 Views	3 Views	4 Views	5 Views
**Borda**	**0.5%**	**0.00015**	**0.00013**	**0.00010**	**0.00009**	**0.00009**
	**1.0%**	**0.00015**	**0.00009**	**0.00009**	**0.00009**	**0.00009**
**TopK**	**0.5%**	**0.00097**	**0.00142**	**0.00012**	**0.00009**	**0.00009**
	**1.0%**	**0.00719**	**0.00037**	**0.00010**	**0.00009**	**0.00009**
**1VI**	**0.5%**	0.48511	**0.01598**	**0.00079**	**0.00079**	**0.00049**
	**1.0%**	*0.31773*	**0.00213**	**0.00053**	**0.00104**	**0.00045**

**Table 2:** Adjusted *p*-values of the Wilcoxon signed rank test performed to compare GENERE with Borda, TopK and 1VI in terms of AUPRC. Statistically significant values (<0.05) are shown in bold. Italic values indicates that the competitor outperforms GENERE. *p*-values are computed by considering the median value over the results obtained with different values of the number of iterations for GENERE, and over the results obtained with different values of k for TopK. Views are obtained with PCA.

GENERE vs	*δ*	1 View	2 Views	3 Views	4 Views	5 Views
**Borda**	**0.5%**	**0.00048**	**0.00048**	**0.00048**	**0.00048**	**0.00048**
	**1.0%**	**0.00052**	**0.00051**	**0.00048**	**0.00052**	**0.00052**
**TopK**	**0.5%**	**0.00052**	**0.00111**	**0.00048**	**0.00048**	**0.00048**
	**1.0%**	**0.00055**	**0.00048**	**0.00048**	**0.00048**	**0.00048**
**1VI**	**0.5%**	0.13240	0.24896	**0.03503**	**0.04408**	**0.04570**
	**1.0%**	*0.14953*	**0.02419**	**0.02130**	**0.04408**	0.05102

**Table 3:** Adjusted *p*-values of the Wilcoxon signed rank test performed to compare GENERE with Borda, TopK and 1VI in terms of AUROC. Statistically significant values (<0.05) are shown in bold. Italic values indicates that the competitor outperforms GENERE. *p*-values are computed by considering the median value over the results obtained with different values of the number of iterations for GENERE, and over the results obtained with different values of k for TopK. Views are obtained with K-means.

GENERE vs	*δ*	1 View	2 Views	3 Views	4 Views	5 Views
**Borda**	**0.5%**	**0.00019**	**0.00025**	**0.00017**	**0.00017**	**0.00017**
	**1.0%**	**0.00020**	**0.00032**	**0.00017**	**0.00017**	**0.00017**
**TopK**	**0.5%**	**0.00075**	**0.00330**	**0.00030**	**0.00017**	**0.00017**
	**1.0%**	**0.00350**	**0.02129**	**0.00034**	**0.00018**	**0.00017**
**1VI**	**0.5%**	0.13085	0.18772	0.05697	**0.00415**	**0.00103**
	**1.0%**	*0.09432*	0.18772	**0.02129**	**0.00125**	**0.00042**

**Table 4:** Adjusted *p*-values of the Wilcoxon signed rank test performed to compare GENERE with Borda, TopK and 1VI in terms of AUPRC. Statistically significant values (<0.05) are shown in bold. Italic values indicates that the competitor outperforms GENERE. *p*-values are computed by considering the median value over the results obtained with different values of the number of iterations for GENERE, and over the results obtained with different values of k for TopK. Views are obtained with K-means.

GENERE vs	*δ*	1 View	2 Views	3 Views	4 Views	5 Views
**Borda**	**0.5%**	**0.00053**	**0.00084**	**0.00053**	**0.00047**	**0.00047**
	**1.0%**	**0.00070**	**0.00070**	**0.00053**	**0.00047**	**0.00047**
**TopK**	**0.5%**	**0.00047**	**0.00557**	**0.00115**	**0.00047**	**0.00045**
	**1.0%**	**0.00055**	**0.00971**	**0.00277**	**0.00047**	**0.00045**
**1VI**	**0.5%**	0.12785	*0.19567*	0.49307	*0.48143*	0.13829
	**1.0%**	***0.02865***	*0.35568*	0.27662	0.11073	**0.02534**

**Table 5:** Average percentage of improvement in terms of AUROC between GENERE (PCA) and the considered competitors (Borda, TopK and 1VI). The results are obtained on the SynTReN datasets and are first grouped by dataset, number of genes and level of noise, then averaged across the different values of *δ*, the number of views *V*, and the number of iterations.

GENERE vs		Noise	100	150	200	Average
**Borda**	**E.coli**	**0.0**	18.44%	38.25%	46.08%	34.26%
		**0.1**	28.60%	42.63%	47.71%	39.64%
		**0.5**	2.13%	20.71%	47.10%	23.31%
	**Yeast**	**0.0**	26.93%	47.26%	66.83%	47.01%
		**0.1**	28.26%	37.58%	63.24%	43.02%
		**0.5**	0.58%	30.25%	37.13%	22.65%
**TBN**	**E.coli**	**0.0**	43.78%	18.19%	41.71%	34.56%
		**0.1**	33.39%	22.73%	42.35%	32.82%
		**0.5**	40.80%	15.96%	33.89%	30.22%
	**Yeast**	**0.0**	2.05%	-3.30%	2.53%	0.43%
		**0.1**	1.88%	-4.67%	5.29%	0.83%
		**0.5**	-4.79%	-5.54%	-0.61%	-3.65%
**1VI**	**E.coli**	**0.0**	10.93%	0.57%	-2.38%	3.04%
		**0.1**	14.96%	3.21%	4.36%	7.51%
		**0.5**	14.42%	18.29%	13.28%	15.33%
	**Yeast**	**0.0**	1.75%	-2.17%	11.65%	3.74%
		**0.1**	10.70%	-0.02%	14.71%	8.47%
		**0.5**	17.15%	17.82%	12.99%	15.99%

**Table 6:** Average percentage of improvement in terms of AUROC between GENERE (K-means) and the considered competitors (Borda, TopK and 1VI). The results are obtained on the SynTReN datasets and are first grouped by dataset, number of genes and level of noise, then averaged across the different values of *δ*, the number of views *V*, and the number of iterations.

GENERE vs		Noise	100	150	200	Average
**Borda**	**E.coli**	**0.0**	68.71%	112.26%	12.11%	64.36%
		**0.1**	61.14%	77.79%	14.39%	51.11%
		**0.5**	7.36%	49.75%	-3.10%	18.01%
	**Yeast**	**0.0**	434.02%	448.11%	216.06%	366.06%
		**0.1**	483.30%	431.42%	162.89%	359.20%
		**0.5**	161.96%	226.62%	92.39%	160.32%
**TopK**	**E.coli**	**0.0**	10.41%	8.08%	12.11%	10.20%
		**0.1**	16.00%	10.29%	10.65%	12.31%
		**0.5**	0.94%	14.91%	-8.22%	2.54%
	**Yeast**	**0.0**	19.83%	19.54%	28.48%	22.62%
		**0.1**	25.86%	26.12%	31.06%	27.68%
		**0.5**	-0.13%	35.91%	19.09%	18.29%
**1VI**	**E.coli**	**0.0**	19.39%	-1.36%	-6.47%	3.85%
		**0.1**	13.60%	-3.82%	15.42%	8.40%
		**0.5**	9.37%	7.82%	-6.42%	3.59%
	**Yeast**	**0.0**	7.24%	-3.91%	2.40%	1.91%
		**0.1**	-2.02%	-0.64%	21.95%	6.43%
		**0.5**	-5.43%	17.60%	9.27%	7.14%

### Analysis of the results

We start by inspecting the boxplots in Figs 4–10 and the figures given in S2 Fig. The first observation we can make is that PCA and K-means lead to similar AUROC and AUPRC results. This confirms that the prformance of our algorithm is generally independent of the clustering algorithm adopted in the generation of the views. However, in some cases (see, for example, *D5Ecoli*), K-means produces slightly better results. This can be explained by the fact that PCA, in its original formulation, is used for soft clustering and not for hard clustering (i.e., generating a partition) that we use it for in our work. On the basis of this observation, in the remainder of our analysis, we mainly focus on the results obtained with views generated by K-means.

Figs 4–10 provide insight into how GENERE compares to its competitors in terms of AUROC and AUPRC. By analyzing these figures, we can draw several conclusions. First, the best values are generally obtained when the number of views is 3, 4 or 5. This means that values of *V* ∈ {3, 4, 5} lead to better groups of base methods that allow us to distinguish better among their properties. This result is in line with Marbach et al. [12], where the authors identified four clusters of base methods. While for the SynTReN datasets the base methods we consider are not the same as those of Marbach et al. [12], the two sets of methods are based on the same main principles.

In S1 Fig, we present the base method clusters identified by PCA and K-means for some of the SynTReN datasets and all the DREAM5 datasets. For the SynTReN datasets the application of PCA does not identify significant differences among the scoring schemes (except for MRNET, which shows a slightly different behavior). As expected, different measures behave differently and our approach recognizes such differences in behavior: Measures that follow the same principles are grouped together. This is true especially for measures operating on vectors and measures based on equal width and equal frequency discretization. In contrast, K-means tends to cluster the base methods more along the scoring schemes than along the different types of measures. For the DREAM5 datasets, PCA seems to better group methods that follow the same principles (see Figs 2 and 3). Despite this difference between the two clustering algorithms we use for the construction of the views, both clustering methods lead to good predictions, confirming that both are valid and that their output is profitably exploited by GENERE.

Second, by comparing the AUROC and AUPRC values obtained for D5inSilico, D5Ecoli and D5Yeast, we note that gene network reconstruction from the D5Yeast dataset produces the worst results (this is true not only for GENERE but also for its competitors). This is possibly due to the base methods used: fundamental assumption of expression-based network inference algorithms is that mRNA levels of regulators and their targets show some degree of mutual dependency. As investigated in Marbach et al. (see Supplementary Notes of [12], Figure S8), in both the D5inSilico and D5Ecoli, mutual dependencies between expression profiles of transcription factors and their known target genes exceed the dependencies observed between non-interacting gene pairs (both in terms of Pearson’s correlation coefficient and mutual information). In D5Yeast (S. cerevisiae), the two dependency distributions are almost identical, suggesting that it is much more difficult to detect transcription factor-gene interactions based on dependencies derived from this compendium of gene expression data used in the DREAM5 network inference challenge.

**Fig 11:**
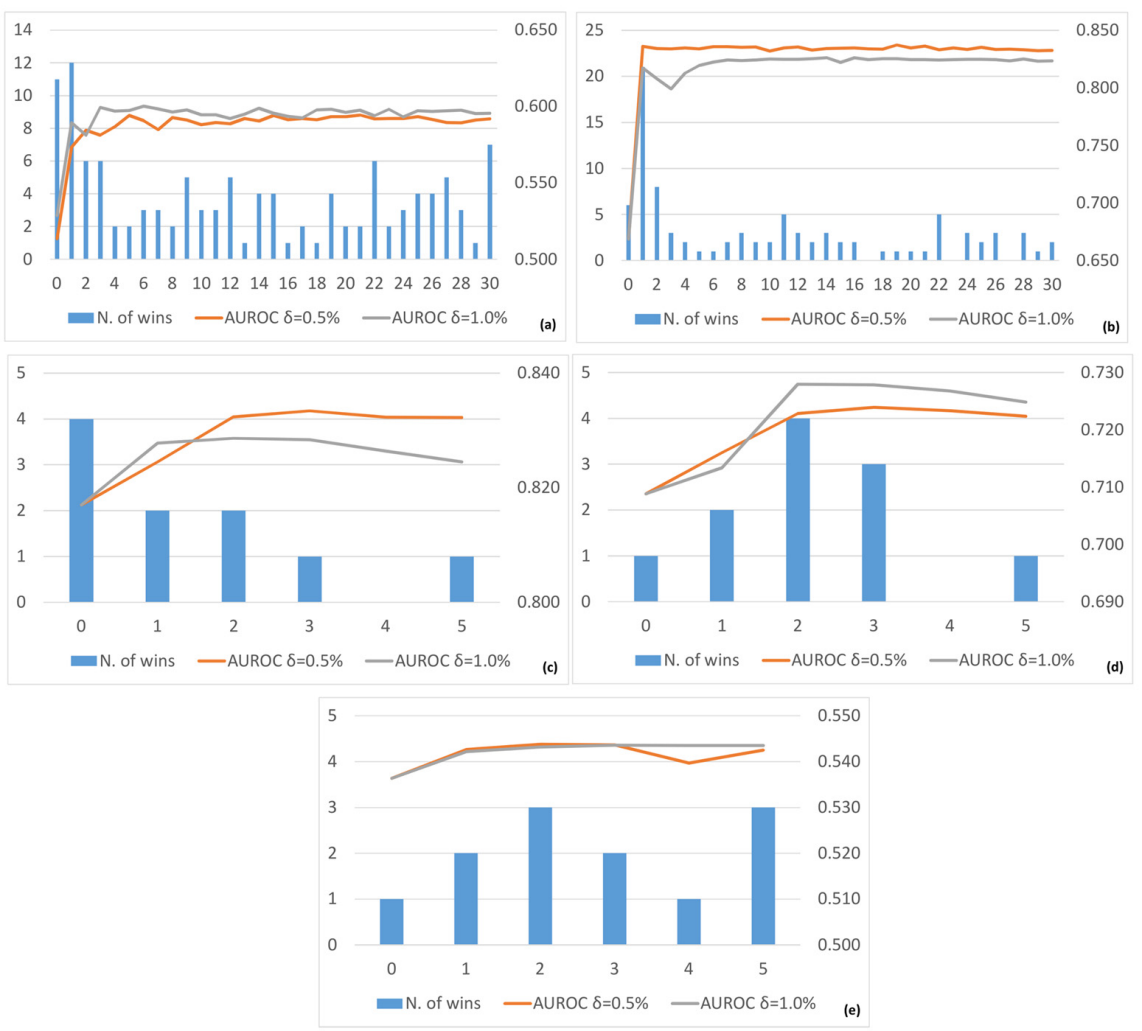
Number of wins, in terms of AUROC (primary *Y*-axis, blue histograms) and average AUROC (secondary *Y*-axis, red and grey lines), obtained for SynTReN *EColi* (a), SynTReN *Yeast* (b), *D5InSilico* (c), *D5Ecoli* (d) and *D5Yeast* (e) obtained by varying the number of iterations (*X*-axis) from 0 to 30 for the SynTReN datasets and from 0 to 5 for the DREAM5 datasets. The red lines represent the average AUROC with *δ* = 0.5%, whereas the gray lines represent the average AUROC with *δ* = 1.0%. Views are obtained with K-means.

**Fig 12:**
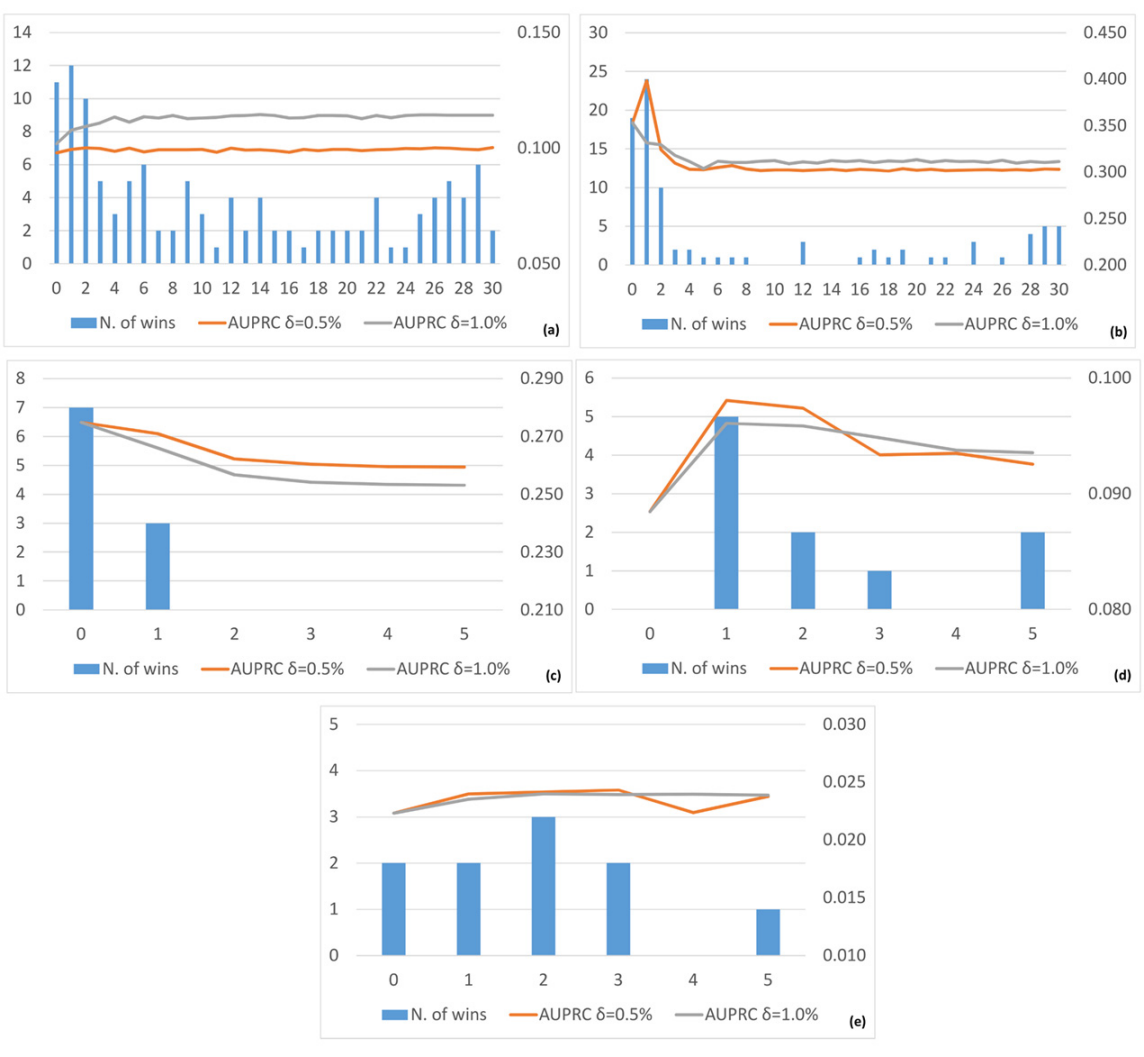
Number of wins, in terms of AUPRC (primary *Y*-axis, blue histograms) and average AUPRC (secondary *Y*-axis, red and grey lines), obtained for SynTReN *EColi* (a), SynTReN *Yeast* (b), *D5InSilico* (c), *D5Ecoli* (d) and *D5Yeast* (e) obtained by varying the number of iterations (*X*-axis) from 0 to 30 for the SynTReN datasets and from 0 to 5 for the DREAM5 datasets. The red lines represent the average AUPRC with *δ* = 0.5%, whereas the gray lines represent the average AUPRC with *δ* = 1.0%. Views are obtained with K-means.

Third, concerning the sensitivity of the GENERE method to the number of iterations, in Fig 11 (resp., in Fig 12), we report a summary of the average AUROC (resp., AUPRC) and the number of wins, i.e., the number of cases for which the considered configuration shows the best AUROC (resp., AUPRC), obtained by varying the number of iterations for each considered dataset. For the SynTReN datasets, the best number of iterations is 2-3 for *E. coli* and 1-2 for *Yeast*, whereas it is 1-2 for all the DREAM5 datasets.

This means that the algorithm converges fast to its best performance with the best number of iterations for each dataset clearly visible from the number of wins. Moreover, the average AUROC and AUPRC generally do not deteriorate when the number of iterations increases beyond the best choice, although the additional iterations do not lead to further improvements.

Fourth, as expected, by increasing the level of noise in the SynTReN datasets, we notice that both the AUROC and AUPRC values decrease. A closer analysis of the results (see Tables 5 and 6) reveals that the gain with respect to Borda and TopK slightly decreases when the level of noise increases, although it remains positive in most cases. However, when the noise becomes very high (i.e., 0.5) our algorithm starts to consider as “reliable” also noisy information, which can be propagated in the subsequent iterations. This is due to the experimental setting and, in particular, to the initialization of *L*. Obviously, we can also use a more informative way for this initialization but, as clarified before, we do not want to give an advantage to our method with respect to its competitors. This problem is also true in 1VI, which uses the same initialization of *L* we use. Indeed, GENERE seems to be more robust to noise than 1VI which does not exploit the iterative multi-view learning approach and is, thus, not able to correct the initial decisions on the basis of different “viewpoints”.

As regards the size of the target network, we can notice that when we use PCA for the construction of the views (see Table 5) increasing the number of nodes generally leads to an increase in the advantage of our approach over the competitors. This means that our algorithm is able to better exploit the information conveyed by the higher number of nodes and interactions in the network. In the case of K-means (see Table 6), the drastic improvements of predictive accuracy over the competitors show less consistent patterns across target networks size.

Furthermore, we applied the Wilcoxon signed rank test with the False Discovery Rate (FDR) correction for multiple tests proposed by Benjamini and Hochberg [39]. Looking at the results in Tables 1–4, we can observe that GENERE significantly outperforms the competitors in terms of median AUROC and AUPRC values. A closer analysis of the *p*-values confirms that the best number of views are generally 3, 4 and 5. Moreover, the fact that GENERE significantly outperforms 1VI (this is evident when the number of views is 5) confirms the advantage of the multi-view learning framework, which can fully exploit the differences between the base methods falling in the same view (i.e., between base methods with similar behavior).

Finally, the results do not show significant changes for different values of *δ* (the percentage of examples considered as positive at each iteration). However, including only more reliable values at each iteration (*δ* = 0.5%) leads to better results (see Tables 3 and 4, lines Borda and TopK). Using a lower value of *δ* is thus advisable. For 1VI, whose results also depend on *δ*, there is no clear and consistent difference in performance for the different values of *δ* across the different number of views.

## Conclusion

In this work, we address the problem of gene regulatory network reconstruction. The solution we propose combines the scores returned by several algorithms for predicting interactions among genes. In contrast to existing approaches, which use very simple combination strategies, we propose a machine learning solution that learns to combine the predictions. The major advantages of our approach are as follows.

First, we use the multi-view learning approach that allows the algorithm to exploit (even small) differences among similar base methods. Second, the views are automatically identified by the system by applying a clustering algorithm. Third, the proposed approach is able to correct decisions taken by some classifiers (learned from a partition of base methods) by exploiting decisions taken by the other classifiers (learned from the other partitions of base methods). Fourth, the proposed approach is able to learn from a small (even empty) set of only positive examples of interactions, thanks to the semi-supervised learning solution that exploits both labeled and unlabeled examples during learning.

Experiments prove that our approach significantly outperforms, in terms of accuracy of reconstructed networks and in different configuration settings, state of the art approaches in the reconstruction of well-known gene interaction networks such as those in *E. coli* and *S. cerevisiae*, from both synthetically generated and real expression data. Improvements are obtained with a small number of iterations, guaranteeing a fast convergence of the algorithm to the best solution. The results indicate that gene regulatory network reconstruction for the real datasets is more difficult for S. cerevisiae than for E. coli.

In further work, we plan to consider gene interactions as inter-dependent and exploit the network autocorrelation among interactions that involve the same/similar genes. Moreover, we intend to investigate new “transfer learning” techniques which exploit GRNs reconstructed from some organisms in the reconstruction of GRNs of other organisms.

## Supporting Information

S1 Text Additional details on the SynTReN and DREAM5 datasets. (PDF)

S1 Fig Graphic representation of views identified with PCA and K-means. (PDF)

S1 Table AUROC/AUPRC results on the SynTReN and DREAM5 datasets. (XLSX)

S2 Table Comparison of AUROC/AUPRC results with different numbers of unlabeled examples for each sample. (XLSX)

S2 Fig AUROC/AUPRC boxplots obtained with PCA-based clustering. (PDF)
