# Supplementary material for: Semi-Supervised Multi-View Learning for Gene Network Reconstruction
Source: PLoS One. 2015 Dec 7;10(12):e0144031. doi: 10.1371/journal.pone.0144031 (PMC4671612; doi:10.1371/journal.pone.0144031)
Supplement: S1 Text — (PDF) [file pone.0144031.s001.pdf]

1 Additional Details on the SynTReN Datasets

The measures considered for the construction of the similarity (or distance) matrix are applied to the generated expression data. They can be categorized into three different groups, based on the nature of the input data structure: measures on vectors, random variables, and symbolic dynamics. Measures operating on vectors include classical distance measures, such as Euclidean, Manhattan and L10Norm [5], and all the three variants (i.e., Asymmetric, Symmetric1 and Symmetric2) of Dynamic Time Warping proposed by [15], leading to a total of 6 measures. Measures operating on random variables are based on correlation coefficients and mutual information. We consider 3 correlation coefficients, that is, Pearson, Spearman and Kendall. Ee compute mutual information with either equal width or equal frequency discretization, by adopting either shrinkage or optimized maximum likelihood bins estimation [22]. Orthogonally, we also investigate the possibility of gene expression profiles to be represented as coarse-grained variables by adopting the Kolmogorov-Sinai entropy to compute mutual information [31]. In sum, by considering all the possible combinations along the three dimensions, we obtain 8 different measures based on mutual information. Finally, measures that operate on symbolic dynamics come from computer science: These are characterized by a qualitative description of gene expression profiles. In our study, we use *1)* the symbolic sequence similarity [10]; *2)* the qualitative distance measure [27]; *3)-4)* the mutual information computed on the symbol vectors by optimized maximum likelihood with either equal frequency or equal width discretization; *5)-6)* the mean between the symbolic sequence similarity and the mutual information (equal frequency/equal width). In summary, we consider 6 measures operating on symbolic dynamics.

As scoring schemes, we used Identity (ID) which does not apply any correction, the Context Likelihood of Relatedness (CLR) [6], the Algorithm for the Reconstruction of Accurate Cellular Networks (ARACNE) [18], the Maximum Relevance/minimum redundancy NETwork (MRNET) [20] and the Asymmetric WEighting (AWE) [10]. Since AWE is able to identify the direction of the regulation, we also considered its use without applying the time shifting technique (AWE w/o TS).

2 Additional Details on the Dream5 Datasets

The scores used by our algorithm are obtained with all the DREAM5 participants' methods. Methods are grouped in six different groups, based on the principle and methodology of scores derivation [17].

Regression methods are based on learning a regression model or a combination of such models. In the context of gene network reconstruction, fitted parameters describe the contribution of each transcription factor in the regulation of the target gene. The learning process is repeated for each gene in the dataset considered as target gene. The core of a method that belongs to this group can employ different approaches for learning or fitting a regression model, with or without resampling. In particular, the considered methods belonging to this category are: *1)* Trustful Inference of Gene REgulation using Stability Selection (TIGRESS) using the Lasso method [26] with resampling, where the regularization parameter selects five transcription factors per target gene in each bootstrap sample [9]; *2)* Combination of steady state and time series data in disjoint groups, using the group Lasso method with bootstrapping [34]; *3)* Combination of Lasso and Bayesian linear regression [21] models learned using Reversible Jump Markov Chain [7] with Monte Carlo simulations [14]; *4)* Lasso method for learning a sparse linear regression model with bootstraping [19]; *5)* Lasso method for learning a sparse linear regression model using area under the stability selection curve for data resampling [19]; *6)* Application of the Lasso toolbox GENLAB using standard parameters [29]; *7)* Lasso models combined by the maximum regularization parameter that selects a given edge for the first time [19]; *8)* Linear regression model that determines the contribution of transcription factors to the expression of target genes [17].

Mutual Information methods work on discretized points of gene expression profiles. First, a discretization step is performed, which is followed by a ranking of the edges based on variants of mutual information. Finally, a filter is used to remove causal relationships with low scores. In particular, the considered methods belonging to this category are: *1)* Context likelihood of relatedness (CLR) with spline estimation of mutual information. Causal relationships are filtered by likelihood score, based on its local network context [6]; *2)* Simple approach that empathizes the discretization of expression values [4]; *3)* Algorithm for the Reconstruction of Accurate Cellular Networks (ARACNE) with kernel estimation of mutual information. The last step consists of data processing inequality to identify direct interactions [18]; *4)* Fast kernel-based estimation of mutual information with Bayesian Local Causal Discovery (BLCD) [16] and Markov blanket (HITON-PC) algorithm [2] to identify direct interactions; *5)* Combination of Pearson correlation coefficient and fast kernel-based estimation of mutual information with Bayesian Local Causal Discovery (BLCD) [16] and Markov blanket (HITON-PC) algorithm [2] to identify direct interactions.

Correlation-based methods exploit variants of correlation coefficients in order to rank the relevance of identified links. The considered methods belonging to this category are: *1)* Absolute value of Pearson's correlation coefficient [4]; *2)* Signed value of Pearson's correlation coefficient [4]; *3)* Signed value of Spearmans correlation coefficient [4].

Bayesian networks. Methods based on this model build a dependency network and optimize the posterior probabilities of existence of each link by exploiting different heuristic searches. The considered methods belonging to this category are: *1)* Bayesian networks using likelihood-based criteria (CATNET), which perform an exhaustive search for fixed node orders and stochastic search of optimal orders via a simulated annealing algorithm, with aggregation of three runs [3]; *2)* Bayesian networks using likelihood-based criteria (CATNET), which are based on an exhaustive search for fixed node orders and stochastic search of optimal orders via simulated annealing algorithm [3]; *3)* Max-Min Parent and Children algorithm (MMPC) with bootstrapped datasets [28]; *4)* Markov blanket algorithm (HITON-PC) [2] with bootstrapped datasets [1]; *5)* Markov boundary induction algorithm (TIE*) with bootstrapped datasets [25]; *6)* Models transcription factor perturbation data and time series using dynamic Bayesian networks [17].

Other approaches that reconstruct networks by exploiting heterogeneous and novel methods. The considered methods belonging to this category are described in the following: *1)* GENIE3: A random forest is trained to predict target gene expression. Putative transcription factors are selected as tree nodes if they consistently reduce the variance of the target [11]; *2)* Co-dependencies between transcription factors and target genes are detected by the non-linear correlation coefficient η^2 (two-way ANOVA). Transcription factor perturbation data are up-weighted [13]; *3)* Transcription factors are selected by maximizing the conditional entropy for target genes, which are represented as Boolean vectors with probabilities, in order to avoid discretization [12]; *4)* Transcription factors are preselected from transcription factor perturbation data or by exploiting Pearson's correlation and then tested by iterative Bayesian Model Averaging (BMA) [32]; *5)* A Gaussian noise model is used to estimate whether the expression of a target gene changes in transcription factor perturbation measurements [33]; *6)* Target genes are clustered by exploiting Pearson's correlation coefficient. A genetic algorithm is adopted to learn the structure of a neural network, whose parameters are learned by exploiting the back-propagation algorithm [23]; *7)* Data are discretized by exploiting Gaussian mixture models and a clustering algorithm (i.e., k-means). Interactions are detected by the generalized logical network modeling (χ^2 test) [24]; *8)* The χ^2 test is applied to evaluate the probability of a shift between transcription factor and target gene expression in transcription factor perturbation experiments [24].

Meta predictors combine different approaches for network reconstruction. In particular, the considered methods belonging to this category consider the following approaches: *1)* Z-scores for target genes in transcription factor knockout data, time-lagged CLR for time series, and linear ordinary differential equation models constrained by Lasso (Inferelator) in combination with a resampling approach [8]; *2)* Pearson's correlation, mutual information and CLR with average ranking aggregation [17]; *3)* Computation of target gene responses in transcription factor knockout data, application of full-order, partial correlation and transcription factor-target co-deviation analysis [17]; *4)* CLR filtered by negative Pearson's correlation coefficient, least angle regression (LARS) of time series, and transcription factor perturbation data and combination by z-scores [30]; *5)* Pearson's correlation coefficient, differential expression (limma) and time series analysis in combination with Naïve Bayes [17].

References

[1] C. F. Aliferis, A. Statnikov, I. Tsamardinos, S. Mani, and X. D. Koutsoukos. Local causal and Markov blanket induction for causal discovery and feature selection for classification - Part I: Algorithms and empirical evaluation. *The Journal of Machine Learning Research*, 11:171–234, 2010.

[2] C. F. Aliferis, I. Tsamardinos, and A. Statnikov. HITON: A novel Markov blanket algorithm for optimal variable selection. *AMIA Ann. Symposium Proceedings*, 2003(1):21–25, 2003.

[3] N. Balov. A categorical network approach for discovering differentially expressed regulations in cancer. *BMC Medical Genomics*, 6(Suppl 3):S1, 2013.

[4] A. J. Butte, P. Tamayo, D. Slonim, T. R. Golub, and I. S. Kohane. Discovering functional relationships between RNA expression and chemotherapeutic susceptibility using relevance networks. *Proceedings of the National Academy of Sciences*, 97(22):12182–12186, 2000.

[5] M. M. Deza and E. Deza. *Encyclopedia of Distances*. Springer, Berlin, 2009.

[6] J. J. Faith, B. Hayete, J. T. Thaden, I. Mogno, J. Wierzbowski, G. Cottarel, S. Kasif, J. J. Collins, and T. S. Gardner. Large-scale mapping and validation of Escherichia coli transcriptional regulation from a compendium of expression profiles. *PLoS Biology*, 5(1):e8+, Jan. 2007.

[7] P. J. Green. Reversible jump Markov chain Monte Carlo computation and Bayesian model determination. *Biometrika*, 82(4):711–732, 1995.

[8] A. Greenfield, A. Madar, H. Ostrer, and R. Bonneau. DREAM4: Combining genetic and dynamic information to identify biological networks and dynamical models. *PloS one*, 5(10):e13397, 2010.

[9] A.-C. Haury, F. Mordelet, P. Vera-Licona, and J.-P. Vert. TIGRESS: Trustful Inference of Gene REgulation using Stability Selection. *BMC Systems Biology*, 6(1):145, 2012.

[10] S. Hempel, A. Koseska, Z. Nikoloski, and J. Kurths. Unraveling gene regulatory networks from time-resolved gene expression data - A measures comparison study. *BMC Bioinformatics*, 12(1):292, 2011.

[11] A. Irrthum, L. Wehenkel, P. Geurts, et al. Inferring regulatory networks from expression data using tree-based methods. *PLoS One*, 5(9):e12776, 2010.

[12] G. Karlebach and R. Shamir. Constructing logical models of gene regulatory networks by integrating transcription factor–DNA interactions with expression data: an entropy-based approach. *Journal of Computational Biology*, 19(1):30–41, 2012.

[13] R. Küffner, T. Petri, P. Tavakkolkhah, L. Windhager, and R. Zimmer. Inferring gene regulatory networks by ANOVA. *Bioinformatics*, 28(10):1376–1382, 2012.

[14] S. Lebre, J. Becq, F. Devaux, M. P. Stumpf, and G. Lelandais. Statistical inference of the time-varying structure of gene-regulation networks. *BMC Systems Biology*, 4(1):130, 2010.

[15] S. Z. Li and A. Jain. Dynamic time warping (DTW). In S. Z. Li and A. Jain, editors, *Encyclopedia of Biometrics*, pages 231–231. Springer New York, 2009.

[16] S. Mani and G. F. Cooper. Causal discovery using a Bayesian local causal discovery algorithm. *Medinfo*, 11(Pt 1):731–735, 2004.

[17] D. Marbach, J. C. Costello, R. Küffner, N. M. Vega, R. J. Prill, D. M. Camacho, K. R. Allison, M. Kellis, J. J. Collins, and G. Stolovitzky. Wisdom of crowds for robust gene network inference. *Nature Methods*, 9:796–804, 2012.

[18] A. Margolin, I. Nemenman, K. Basso, C. Wiggins, G. Stolovitzky, R. Favera, and A. Califano. ARACNE: An algorithm for the reconstruction of gene regulatory networks in a mammalian cellular context. *BMC Bioinformatics*, 7(Suppl 1):S7, 2006.

[19] N. Meinshausen and P. Bühlmann. Stability selection. *Journal of the Royal Statistical Society: Series B (Statistical Methodology)*, 72(4):417–473, 2010.

[20] P. E. Meyer, K. Kontos, F. Lafitte, and G. Bontempi. Information-theoretic inference of large transcriptional regulatory networks. *EURASIP Journal on Bioinformatics and Systems Biology*, 2007.

[21] T. Minka. Bayesian linear regression. Technical report, MIT Media Lab note, 2000.

[22] A. Papoulis and S. Pillai. *Probability, Random Variables, and Stochastic Processes.* Tata McGraw-Hill, 2002.

[23] A. Sîrbu, H. J. Ruskin, and M. Crane. *Stages of Gene Regulatory Network Inference: The Evolutionary Algorithm Role.* INTECH Open Access Publisher, Rijeka, 2011.

[24] M. J. Song, C. K. Lewis, E. R. Lance, E. J. Chesler, R. K. Yordanova, M. A. Langston, K. H. Lodowski, and S. E. Bergeson. Reconstructing generalized logical networks of transcriptional regulation in mouse brain from temporal gene expression data. *EURASIP Journal Bioinform. Syst. Biol*, 2009:545176, 2009.

[25] A. Statnikov and C. F. Aliferis. Analysis and computational dissection of molecular signature multiplicity. *PLoS Computational Biology*, 6(5):e1000790, 2010.

[26] R. Tibshirani. Regression shrinkage and selection via the Lasso. *Journal of the Royal Statistical Society. Series B (Methodological)*, pages 267–288, 1996.

[27] L. Todorovski, B. Cestnik, and M. Kline. Qualitative clustering of short time-series: A case study of firms reputation data. *International Workshop on Integration and Collaboration Aspects of Data Mining, Decision Support and Meta-Learning*, page 141, 2002.

[28] I. Tsamardinos, C. F. Aliferis, and A. Statnikov. Time and sample efficient discovery of Markov blankets and direct causal relations. In *Proceedings of the 9th ACM SIGKDD International Conference on Knowledge Discovery and Data Mining*, pages 673–678. ACM NY, 2003.

[29] E. P. van Someren, B. L. Vaes, W. T. Steegenga, A. M. Sijbers, K. J. Dechering, and M. J. Reinders. Least absolute regression network analysis of the murine osteoblast differentiation network. *Bioinformatics*, 22(4):477–484, 2006.

[30] J. Watkinson, K.-c. Liang, X. Wang, T. Zheng, and D. Anastassiou. Inference of regulatory gene interactions from expression data using three-way mutual information. *Annals of the New York Academy of Sciences*, 1158(1):302–313, 2009.

[31] B. D. Wissman, L. C. McKay-Jones, and P.-M. Binder. Entropy rate estimates from mutual information. *Physical Review E*, 84:046204, Oct 2011.

[32] K. Y. Yeung, R. E. Bumgarner, and A. E. Raftery. Bayesian model averaging: Development of an improved multi-class, gene selection and classification tool for microarray data. *Bioinformatics*, 21(10):2394–2402, 2005.

[33] K. Y. Yip, R. P. Alexander, K.-K. Yan, and M. Gerstein. Improved reconstruction of in silico gene regulatory networks by integrating knockout and perturbation data. *PloS One*, 5(1):e8121, 2010.

[34] M. Yuan and Y. Lin. Model selection and estimation in regression with grouped variables. *Journal of the Royal Statistical Society: Series B (Statistical Methodology)*, 68(1):49–67, 2006.
