# Supplementary material for: Semi-Supervised Multi-View Learning for Gene Network Reconstruction
Source: PLoS One. 2015 Dec 7;10(12):e0144031. doi: 10.1371/journal.pone.0144031 (PMC4671612; doi:10.1371/journal.pone.0144031)
Supplement: S1 Fig — (PDF) [file pone.0144031.s002.pdf]

Results with PCA clustering

Measures	Scoring Schemes					
	ARACNE	AWE	AWE w/o TF	CLR	ID	MRNET
Dynamic Time Warping (Asymmetric constraint path)	2	2	2	2	2	2
Dynamic Time Warping (Symmetric constraint path 1)	2	2	2	2	2	2
Dynamic Time Warping (Symmetric constraint path 2)	2	2	2	2	2	2
Euclidian distance	2	2	2	2	2	2
L10Norm distance	2	2	2	2	2	2
Manhattan distance	2	2	2	2	2	2
Kendall correlation coefficient	1	3	3	3	1	3
MI - Equal Frequency discretization through optimized maximum likelihood	1	1	1	3	1	3
MI - Equal Frequency discretization through optimized maximum likelihood over coarse-grained variables (Kolmogorov-Sinai entropy)				3		3
MI - Equal Frequency discretization through shrinkage	1	1	1	1	1	3
MI - Equal Frequency discretization through shrinkage over coarse-grained variables (Kolmogorov-Sinai entropy)						3
MI - Equal Width discretization through optimized maximum likelihood	1	1	1	1	1	1
MI - Equal Width discretization through optimized maximum likelihood over coarse-grained variables (Kolmogorov-Sinai entropy)	2	3	3	2	3	2
MI - Equal Width discretization through shrinkage	1	1	1	1	1	1
MI - Equal Width discretization through shrinkage over coarse-grained variables (Kolmogorov-Sinai entropy)						3
Pearson correlation coefficient	3	1	1	3	1	1
Spearman correlation coefficient	1	3	3	3	1	3
Mean between symbolic sequence similarity and MI on symbol vectors with Equal Frequency discretization through optimized maximum likelihood	1	1	1	1	1	3
Mean between symbolic sequence similarity and MI on symbol vectors with Equal Width discretization through optimized maximum likelihood	1	1	1	1	1	3
MI on symbol vectors with Equal Frequency discretization through optimized maximum likelihood	1	1	1	1	1	3
MI on symbol vectors with Equal Width discretization through optimized maximum likelihood	3	3	3	1	3	3
Qualitative distance measure	3	3	3	3	3	3
Symbol sequence similarity	1	3	3	3	1	3

Figure 1: Clustering (PCA) of base methods into 3 views (clusters) for the SynTReN *E. coli* dataset with 100 nodes and noise level 0.0. Each base method is a combination of a distance measure and a scoring scheme (cf. Supplementary File 1). The number (and the corresponding color) in a cell identifies the cluster to which a base method is assigned. Empty cells indicate that the corresponding base method has not been associated with any cluster. This can happen if the base method returns no interactions.

Measures	Scoring Schemes					
	ARACNE	AWE	AWE w/o TF	CLR	ID	MRNET
Dynamic Time Warping (Asymmetric constraint path)	2	2	2	2	2	2
Dynamic Time Warping (Symmetric constraint path 1)	2	2	2	2	2	2
Dynamic Time Warping (Symmetric constraint path 2)	2	2	2	2	2	2
Euclidian distance	2	2	2	2	2	2
L10Norm distance	2	2	2	2	2	2
Manhattan distance	2	2	2	2	2	2
Kendall correlation coefficient	1	3	3	3	1	4
MI - Equal Frequency discretization through optimized maximum likelihood	1	1	1	3	1	3
MI - Equal Frequency discretization through optimized maximum likelihood over coarse-grained variables (Kolmogorov-Sinai entropy)				3		4
MI - Equal Frequency discretization through shrinkage	1	1	1	1	1	3
MI - Equal Frequency discretization through shrinkage over coarse-grained variables (Kolmogorov-Sinai entropy)						4
MI - Equal Width discretization through optimized maximum likelihood	4	4	4	4	4	4
MI - Equal Width discretization through optimized maximum likelihood over coarse-grained variables (Kolmogorov-Sinai entropy)	4	3	3	4	3	4
MI - Equal Width discretization through shrinkage	4	4	4	4	4	4
MI - Equal Width discretization through shrinkage over coarse-grained variables (Kolmogorov-Sinai entropy)						4
Pearson correlation coefficient	3	1	1	1	1	3
Spearman correlation coefficient	1	3	3	3	1	4
Mean between symbolic sequence similarity and MI on symbol vectors with Equal Frequency discretization through optimized maximum likelihood	1	1	1	1	1	4
Mean between symbolic sequence similarity and MI on symbol vectors with Equal Width discretization through optimized maximum likelihood	1	1	1	1	1	4
MI on symbol vectors with Equal Frequency discretization through optimized maximum likelihood	1	1	1	1	1	4
MI on symbol vectors with Equal Width discretization through optimized maximum likelihood	3	3	3	4	3	4
Qualitative distance measure	3	4	4	3	4	4
Symbol sequence similarity	1	3	3	3	1	3

Figure 2: Clustering (PCA) of base methods into 4 views (clusters) for the SynTReN *E. coli* dataset with 100 nodes and noise level 0.0. Each base method is a combination of a distance measure and a scoring scheme (cf. Supplementary File 1). The number (and the corresponding color) in a cell identifies the cluster to which a base method is assigned. Empty cells indicate that the corresponding base method has not been associated with any cluster. This can happen if the base method returns no interactions.

Measures	Scoring Schemes					
	ARACNE	AWE	AWE w/o TF	CLR	ID	MRNET
Dynamic Time Warping (Asymmetric constraint path)	2	2	2	2	2	2
Dynamic Time Warping (Symmetric constraint path 1)	2	2	2	2	2	2
Dynamic Time Warping (Symmetric constraint path 2)	2	2	2	2	2	2
Euclidian distance	2	2	2	2	2	2
Kendall correlation coefficient	1	5	5	5	1	3
L10Norm distance	2	1	1	2	1	2
Manhattan distance	2	2	2	2	2	2
Mean between symbolic sequence similarity and MI on symbol vectors with Equal Frequency discretization through optimized maximum likelihood	1	1	1	5	1	3
Mean between symbolic sequence similarity and MI on symbol vectors with Equal Width discretization through optimized maximum likelihood	4	1	1	5	1	3
MI - Equal Frequency discretization through optimized maximum likelihood	1	1	1	5	1	3
MI - Equal Frequency discretization through optimized maximum likelihood over coarse-grained variables (Kolmogorov-Sinai entropy)				5		3
MI - Equal Frequency discretization through shrinkage	1	1	1	5	1	3
MI - Equal Frequency discretization through shrinkage over coarse-grained variables (Kolmogorov-Sinai entropy)						3
MI - Equal Width discretization through optimized maximum likelihood	5	4	4	4	4	5
MI - Equal Width discretization through optimized maximum likelihood over coarse-grained variables (Kolmogorov-Sinai entropy)	2	4	4	4	4	2
MI - Equal Width discretization through shrinkage	4	4	4	4	4	5
MI - Equal Width discretization through shrinkage over coarse-grained variables (Kolmogorov-Sinai entropy)						3
MI on symbol vectors with Equal Frequency discretization through optimized maximum likelihood	1	1	1	5	1	3
MI on symbol vectors with Equal Width discretization through optimized maximum likelihood	4	1	1	5	4	3
Pearson correlation coefficient	4	4	4	4	1	4
Qualitative distance measure	4	4	4	4	4	3
Spearman correlation coefficient	1	5	5	5	1	3
Symbol sequence similarity	4	5	5	5	1	3

Figure 3: Clustering (PCA) of base methods into 5 views (clusters) for the SynTReN *E. coli* dataset with 100 nodes and noise level 0.0. Each base method is a combination of a distance measure and a scoring scheme (cf. Supplementary File 1). The number (and the corresponding color) in a cell identifies the cluster to which a base method is assigned. Empty cells indicate that the corresponding base method has not been associated with any cluster. This can happen if the base method returns no interactions.

Measures	Scoring Schemes					
	ARACNE	AWE	AWE w/o TF	CLR	ID	MRNET
Dynamic Time Warping (Asymmetric constraint path)	2	2	2	2	2	2
Dynamic Time Warping (Symmetric constraint path 1)	2	2	2	2	2	2
Dynamic Time Warping (Symmetric constraint path 2)	2	2	2	2	2	2
Euclidian distance	2	2	2	2	2	2
L10Norm distance	2	1	1	2	2	2
Manhattan distance	2	2	2	2	2	2
Kendall correlation coefficient	1	3	3	3	1	3
MI - Equal Frequency discretization through optimized maximum likelihood	1	1	1	1	1	3
MI - Equal Frequency discretization through optimized maximum likelihood over coarse-grained variables (Kolmogorov-Sinai entropy)				3		3
MI - Equal Frequency discretization through shrinkage	1	1	1	1	1	3
MI - Equal Frequency discretization through shrinkage over coarse-grained variables (Kolmogorov-Sinai entropy)						3
MI - Equal Width discretization through optimized maximum likelihood	3	1	1	1	3	1
MI - Equal Width discretization through optimized maximum likelihood over coarse-grained variables (Kolmogorov-Sinai entropy)	2	2	2	3	2	2
MI - Equal Width discretization through shrinkage	3	1	1	1	3	1
MI - Equal Width discretization through shrinkage over coarse-grained variables (Kolmogorov-Sinai entropy)						3
Pearson correlation coefficient	1	1	1	3	1	1
Spearman correlation coefficient	1	3	3	3	1	3
Mean between symbolic sequence similarity and MI on symbol vectors with Equal Frequency discretization through optimized maximum likelihood	1	1	1	3	1	3
Mean between symbolic sequence similarity and MI on symbol vectors with Equal Width discretization through optimized maximum likelihood	1	1	1	3	1	3
MI on symbol vectors with Equal Frequency discretization through optimized maximum likelihood	1	1	1	3	1	3
MI on symbol vectors with Equal Width discretization through optimized maximum likelihood	1	1	1	1	1	3
Qualitative distance measure	1	3	3	1	3	3
Symbol sequence similarity	1	3	3	3	1	3

Figure 4: Clustering (PCA) of base methods into 3 views (clusters) for the SynTReN *Yeast* dataset with 100 nodes and noise level 0.0. Each base method is a combination of a distance measure and a scoring scheme (cf. Supplementary File 1). The number (and the corresponding color) in a cell identifies the cluster to which a base method is assigned. Empty cells indicate that the corresponding base method has not been associated with any cluster. This can happen if the base method returns no interactions.

Measures	Scoring Schemes					
	ARACNE	AWE	AWE w/o TF	CLR	ID	MRNET
Dynamic Time Warping (Asymmetric constraint path)	2	2	2	2	2	2
Dynamic Time Warping (Symmetric constraint path 1)	2	2	2	2	2	2
Dynamic Time Warping (Symmetric constraint path 2)	2	2	2	2	2	2
Euclidian distance	2	2	2	2	2	2
L10Norm distance	2	2	2	2	2	2
Manhattan distance	2	2	2	2	2	2
Kendall correlation coefficient	1	3	3	3	1	4
MI - Equal Frequency discretization through optimized maximum likelihood	1	1	1	3	1	3
MI - Equal Frequency discretization through optimized maximum likelihood over coarse-grained variables (Kolmogorov-Sinai entropy)				3		4
MI - Equal Frequency discretization through shrinkage	1	1	1	1	1	3
MI - Equal Frequency discretization through shrinkage over coarse-grained variables (Kolmogorov-Sinai entropy)						4
MI - Equal Width discretization through optimized maximum likelihood	4	4	4	4	4	4
MI - Equal Width discretization through optimized maximum likelihood over coarse-grained variables (Kolmogorov-Sinai entropy)	4	3	3	4	3	4
MI - Equal Width discretization through shrinkage	4	4	4	4	4	4
MI - Equal Width discretization through shrinkage over coarse-grained variables (Kolmogorov-Sinai entropy)						4
Pearson correlation coefficient	3	1	1	1	1	3
Spearman correlation coefficient	1	3	3	3	1	4
Mean between symbolic sequence similarity and MI on symbol vectors with Equal Frequency discretization through optimized maximum likelihood	1	1	1	1	1	4
Mean between symbolic sequence similarity and MI on symbol vectors with Equal Width discretization through optimized maximum likelihood	1	1	1	1	1	4
MI on symbol vectors with Equal Frequency discretization through optimized maximum likelihood	1	1	1	1	1	4
MI on symbol vectors with Equal Width discretization through optimized maximum likelihood	3	3	3	4	3	4
Qualitative distance measure	3	4	4	3	4	4
Symbol sequence similarity	1	3	3	3	1	3

Figure 5: Clustering (PCA) of base methods into 4 views (clusters) for the SynTReN *Yeast* dataset with 100 nodes and noise level 0.0. Each base method is a combination of a distance measure and a scoring scheme (cf. Supplementary File 1). The number (and the corresponding color) in a cell identifies the cluster to which a base method is assigned. Empty cells indicate that the corresponding base method has not been associated with any cluster. This can happen if the base method returns no interactions.

Measures	Scoring Schemes					
	ARACNE	AWE	AWE w/o TF	CLR	ID	MRNET
Dynamic Time Warping (Asymmetric constraint path)	2	2	2	2	2	2
Dynamic Time Warping (Symmetric constraint path 1)	2	2	2	2	2	2
Dynamic Time Warping (Symmetric constraint path 2)	2	2	2	2	2	2
Euclidian distance	2	5	5	2	2	2
Kendall correlation coefficient	1	4	4	4	1	3
L10Norm distance	2	5	5	2	1	2
Manhattan distance	2	2	2	2	2	2
Mean between symbolic sequence similarity and MI on symbol vectors with Equal Frequency discretization through optimized maximum likelihood	4	1	1	4	1	3
Mean between symbolic sequence similarity and MI on symbol vectors with Equal Width discretization through optimized maximum likelihood	4	1	1	4	4	3
MI - Equal Frequency discretization through optimized maximum likelihood	5	5	5	4	5	3
MI - Equal Frequency discretization through optimized maximum likelihood over coarse-grained variables (Kolmogorov-Sinai entropy)				2		4
MI - Equal Frequency discretization through shrinkage	5	5	5	4	5	3
MI - Equal Frequency discretization through shrinkage over coarse-grained variables (Kolmogorov-Sinai entropy)						4
MI - Equal Width discretization through optimized maximum likelihood	3	3	3	3	3	5
MI - Equal Width discretization through optimized maximum likelihood over coarse-grained variables (Kolmogorov-Sinai entropy)	5	2	2	4	2	5
MI - Equal Width discretization through shrinkage	3	3	3	3	3	5
MI - Equal Width discretization through shrinkage over coarse-grained variables (Kolmogorov-Sinai entropy)						4
MI on symbol vectors with Equal Frequency discretization through optimized maximum likelihood	4	1	1	4	4	3
MI on symbol vectors with Equal Width discretization through optimized maximum likelihood	4	1	1	1	4	3
Pearson correlation coefficient	3	3	3	4	1	3
Qualitative distance measure	4	5	5	4	5	3
Spearman correlation coefficient	1	4	4	4	1	3
Symbol sequence similarity	5	4	4	4	5	3

Figure 6: Clustering (PCA) of base methods into 5 views (clusters) for the SynTReN *Yeast* dataset with 100 nodes and noise level 0.0. Each base method is a combination of a distance measure and a scoring scheme (cf. Supplementary File 1). The number (and the corresponding color) in a cell identifies the cluster to which a base method is assigned. Empty cells indicate that the corresponding base method has not been associated with any cluster. This can happen if the base method returns no interactions.

D5InSilico - 3 Views

Methods	
Regression1	1
Regression5	1
Other1	1
Other3	1
Meta4	1
Regression3	1
Regression6	1
Regression8	1
MutualInformation1	1
MutualInformation3	1
Correlation1	1
Other4	1
Meta2	1
Meta3	1
Regression2	2
Regression4	2
Regression7	2
MutualInformation2	2
MutualInformation4	2
Bayesian4	2
Bayesian5	2
Other2	2
Other5	2
Other6	3
Meta1	3
MutualInformation5	3
Correlation2	3
Correlation3	3
Other8	3
Bayesian1	3
Bayesian2	3
Bayesian3	3
Bayesian6	3
Other7	3
Meta5	3

D5InSilico - 4 Views

Methods	
Correlation1	1
Meta2	1
Meta4	1
MutualInformation1	1
MutualInformation3	1
Other1	1
Other3	1
Other4	1
Regression1	1
Regression3	1
Regression5	1
Regression6	1
Regression8	1
Bayesian4	2
Bayesian5	2
MutualInformation2	2
MutualInformation4	2
Other2	2
Regression2	2
Regression4	2
Bayesian1	3
Bayesian2	3
Bayesian3	3
Correlation2	3
Correlation3	3
Meta1	3
Meta5	3
MutualInformation5	3
Other6	3
Other7	3
Other8	3
Bayesian6	4
Meta3	4
Other5	4
Regression7	4

D5InSilico - 5 Views

Methods	
Meta2	1
Correlation1	1
Meta4	1
MutualInformation1	1
Other4	1
Regression3	1
Other1	1
Other3	1
Regression5	1
Regression1	1
Bayesian5	2
Bayesian4	2
Regression4	2
Other2	2
Regression2	2
Other8	3
Other7	3
Bayesian1	3
MutualInformation5	3
Bayesian2	3
Meta5	3
Other6	3
Bayesian3	3
Meta1	3
Bayesian6	4
Meta3	4
Other5	4
Regression7	4
Regression8	5
Correlation2	5
MutualInformation2	5
Correlation3	5
MutualInformation3	5
Regression6	5
MutualInformation4	5

D5EColi - 3 Views

Methods	
Correlation1	1
Correlation2	1
Correlation3	1
Meta1	1
Meta2	1
Meta4	1
MutualInformation1	1
Other1	1
Other3	1
Regression1	1
Meta3	2
MutualInformation2	2
MutualInformation3	2
MutualInformation4	2
MutualInformation5	2
Other4	2
Other6	2
Other7	2
Other8	2
Regression2	2
Regression3	2
Regression4	2
Regression5	2
Regression6	2
Regression8	2
Bayesian1	3
Bayesian2	3
Bayesian3	3
Bayesian4	3
Bayesian5	3
Bayesian6	3
Meta5	3
Other2	3
Other5	3
Regression7	3

D5EColi - 4 Views

Methods	
Regression1	1
Other1	1
Other3	1
Meta1	1
Meta4	1
MutualInformation1	1
Correlation1	1
Correlation2	1
Correlation3	1
Meta2	1
Regression5	2
Other6	2
Regression2	2
Regression3	2
Regression4	2
Regression6	2
Regression8	2
MutualInformation2	2
MutualInformation3	2
MutualInformation4	2
MutualInformation5	2
Bayesian1	3
Bayesian2	3
Bayesian3	3
Bayesian4	3
Bayesian5	3
Bayesian6	3
Regression7	4
Other2	4
Other4	4
Other5	4
Other7	4
Other8	4
Meta3	4
Meta5	4

D5EColi - 5 Views

Methods	
Other3	1
Correlation1	1
MutualInformation1	1
Correlation2	1
Meta2	1
Meta4	1
Regression1	1
Correlation3	1
Meta1	1
Other1	1
Regression5	2
Regression4	2
MutualInformation5	2
Regression2	2
Regression8	2
MutualInformation2	2
MutualInformation4	2
MutualInformation3	2
Regression6	2
Other6	2
Bayesian4	3
Bayesian5	3
Bayesian6	3
Bayesian3	3
Other4	4
Other2	4
Other5	4
Regression7	4
Other8	4
Meta3	4
Other7	4
Meta5	4
Bayesian1	5
Bayesian2	5
Regression3	5

D5Yeast - 3 Views

Methods	
Bayesian3	1
Bayesian6	1
Correlation1	1
Correlation2	1
Meta2	1
Meta4	1
MutualInformation1	1
MutualInformation5	1
Other1	1
Other3	1
Other4	1
Other6	1
Regression1	1
Correlation3	2
Meta3	2
MutualInformation2	2
MutualInformation3	2
MutualInformation4	2
Other5	2
Other7	2
Other8	2
Regression3	2
Regression4	2
Regression5	2
Regression6	2
Regression7	2
Regression8	2
Bayesian1	3
Bayesian2	3
Bayesian4	3
Bayesian5	3
Meta1	3
Meta5	3
Other2	3
Regression2	3

D5Yeast - 4 Views

Methods	
Bayesian3	1
Correlation1	1
Correlation2	1
Meta2	1
MutualInformation1	1
MutualInformation5	1
Other1	1
Other3	1
Other4	1
Regression1	1
Correlation3	2
Meta3	2
MutualInformation2	2
MutualInformation3	2
MutualInformation4	2
Other8	2
Regression3	2
Regression4	2
Regression5	2
Regression6	2
Regression8	2
Bayesian1	3
Bayesian2	3
Bayesian4	4
Bayesian5	4
Bayesian6	4
Meta1	4
Meta4	4
Meta5	4
Other2	4
Other5	4
Other6	4
Other7	4
Regression2	4
Regression7	4

D5Yeast - 5 Views

Methods	
MutualInformation5	1
Other1	1
Correlation2	1
Correlation1	1
Other4	1
MutualInformation1	1
Other3	1
Meta2	1
Bayesian3	1
Regression1	1
MutualInformation4	2
Regression4	2
MutualInformation2	2
Regression3	2
Regression8	2
Regression5	2
Regression6	2
Correlation3	2
MutualInformation3	2
Bayesian1	3
Bayesian2	3
Regression2	4
Bayesian5	4
Meta1	4
Bayesian4	4
Bayesian6	4
Other6	4
Other2	5
Meta3	5
Other8	5
Regression7	5
Meta4	5
Other7	5
Other5	5
Meta5	5

Figure 7: Clustering (PCA) of base methods for the DREAM5 datasets into 3, 4 and 5 views (clusters). The number (and the corresponding color) in a cell identifies the cluster to which a base method is assigned.

Results with K-means clustering

Measures	Scoring Schemes					
	ARACNE	AWE	AWE w/o TF	CLR	ID	MRNET
Dynamic Time Warping (Asymmetric constraint path)	2	2	2	2	2	2
Dynamic Time Warping (Symmetric constraint path 1)	2	2	2	2	2	2
Dynamic Time Warping (Symmetric constraint path 2)	2	2	2	2	2	2
Euclidian distance	2	2	2	2	2	2
Kendall correlation coefficient	3	2	2	2	3	2
L10Norm distance	2	2	2	2	2	2
Manhattan distance	2	2	2	2	2	2
Mean between symbolic sequence similarity and MI on symbol vectors with Equal Frequency discretization through optimized maximum likelihood	3	2	2	2	3	2
Mean between symbolic sequence similarity and MI on symbol vectors with Equal Width discretization through optimized maximum likelihood	3	2	2	2	3	2
MI - Equal Frequency discretization through optimized maximum likelihood	3	2	2	2	3	2
MI - Equal Frequency discretization through optimized maximum likelihood over coarse-grained variables (Kolmogorov-Sinai entropy)	1	2	2	2	1	2
MI - Equal Frequency discretization through shrinkage	3	2	2	2	3	2
MI - Equal Frequency discretization through shrinkage over coarse-grained variables (Kolmogorov-Sinai entropy)	1	2	2		1	2
MI - Equal Width discretization through optimized maximum likelihood	2	2	2	2	3	2
MI - Equal Width discretization through optimized maximum likelihood over coarse-grained variables (Kolmogorov-Sinai entropy)	2	2	2	2	1	2
MI - Equal Width discretization through shrinkage	2	2	2	2	3	2
MI - Equal Width discretization through shrinkage over coarse-grained variables (Kolmogorov-Sinai entropy)	1	2	2		1	2
MI on symbol vectors with Equal Frequency discretization through optimized maximum likelihood	3	2	2	2	3	2
MI on symbol vectors with Equal Width discretization through optimized maximum likelihood	3	2	2	2	3	2
Pearson correlation coefficient	2	2	2	2	2	2
Qualitative distance measure	3	2	2	3	1	2
Spearman correlation coefficient	3	2	2	2	3	2
Symbol sequence similarity	3	2	2	2	3	2

Figure 8: Clustering (K-means) of base methods into 3 views (clusters) for the SynTReN *E. coli* dataset with 100 nodes and noise level 0.0. Each base method is a combination of a distance measure and a scoring scheme (cf. Supplementary File 1). The number (and the corresponding color) in a cell identifies the cluster to which a base method is assigned. Empty cells indicate that the corresponding base method has not been associated with any cluster. This can happen if the base method returns no interactions.

Measures	Scoring Schemes					
	ARACNE	AWE	AWE w/o TF	CLR	ID	MRNET
Dynamic Time Warping (Asymmetric constraint path)	4	2	2	2	4	4
Dynamic Time Warping (Symmetric constraint path 1)	4	2	2	4	4	4
Dynamic Time Warping (Symmetric constraint path 2)	4	2	2	2	4	4
Euclidian distance	4	2	2	4	4	4
Kendall correlation coefficient	3	2	2	2	3	2
L10Norm distance	4	2	2	2	4	4
Manhattan distance	4	2	2	4	4	4
Mean between symbolic sequence similarity and MI on symbol vectors with Equal Frequency discretization through optimized maximum likelihood	3	2	2	2	3	2
Mean between symbolic sequence similarity and MI on symbol vectors with Equal Width discretization through optimized maximum likelihood	3	2	2	2	3	2
MI - Equal Frequency discretization through optimized maximum likelihood	3	2	2	2	3	2
MI - Equal Frequency discretization through optimized maximum likelihood over coarse-grained variables (Kolmogorov-Sinai entropy)	1	2	2	2	1	2
MI - Equal Frequency discretization through shrinkage	3	2	2	2	3	2
MI - Equal Frequency discretization through shrinkage over coarse-grained variables (Kolmogorov-Sinai entropy)	1	2	2		1	2
MI - Equal Width discretization through optimized maximum likelihood	2	2	2	2	3	2
MI - Equal Width discretization through optimized maximum likelihood over coarse-grained variables (Kolmogorov-Sinai entropy)	2	2	2	2	1	2
MI - Equal Width discretization through shrinkage	2	2	2	2	3	2
MI - Equal Width discretization through shrinkage over coarse-grained variables (Kolmogorov-Sinai entropy)	1	2	2		1	2
MI on symbol vectors with Equal Frequency discretization through optimized maximum likelihood	3	2	2	2	3	2
MI on symbol vectors with Equal Width discretization through optimized maximum likelihood	3	2	2	2	3	2
Pearson correlation coefficient	2	2	2	2	2	2
Qualitative distance measure	3	2	2	3	1	2
Spearman correlation coefficient	3	2	2	2	3	2
Symbol sequence similarity	3	2	2	2	3	2

Figure 9: Clustering (K-means) of base methods into 4 views (clusters) for the SynTReN *E. coli* dataset with 100 nodes and noise level 0.0. Each base method is a combination of a distance measure and a scoring scheme (cf. Supplementary File 1). The number (and the corresponding color) in a cell identifies the cluster to which a base method is assigned. Empty cells indicate that the corresponding base method has not been associated with any cluster. This can happen if the base method returns no interactions.

Measures	Scoring Schemes					
	ARACNE	AWE	AWE w/o TF	CLR	ID	MRNET
Dynamic Time Warping (Asymmetric constraint path)	4	2	2	2	4	4
Dynamic Time Warping (Symmetric constraint path 1)	4	2	2	2	4	4
Dynamic Time Warping (Symmetric constraint path 2)	4	2	2	2	4	4
Euclidian distance	4	2	2	4	4	4
Kendall correlation coefficient	5	2	2	2	5	3
L10Norm distance	4	2	2	2	4	4
Manhattan distance	4	2	2	4	4	4
Mean between symbolic sequence similarity and MI on symbol vectors with Equal Frequency discretization through optimized maximum likelihood	5	2	2	2	5	3
Mean between symbolic sequence similarity and MI on symbol vectors with Equal Width discretization through optimized maximum likelihood	5	2	2	2	5	3
MI - Equal Frequency discretization through optimized maximum likelihood	5	2	2	2	5	3
MI - Equal Frequency discretization through optimized maximum likelihood over coarse-grained variables (Kolmogorov-Sinai entropy)	1	2	2	2	1	2
MI - Equal Frequency discretization through shrinkage	5	2	2	2	5	3
MI - Equal Frequency discretization through shrinkage over coarse-grained variables (Kolmogorov-Sinai entropy)	1	2	2		1	2
MI - Equal Width discretization through optimized maximum likelihood	3	2	2	2	3	2
MI - Equal Width discretization through optimized maximum likelihood over coarse-grained variables (Kolmogorov-Sinai entropy)	2	2	2	2	1	2
MI - Equal Width discretization through shrinkage	3	2	2	2	3	2
MI - Equal Width discretization through shrinkage over coarse-grained variables (Kolmogorov-Sinai entropy)	1	2	2		1	2
MI on symbol vectors with Equal Frequency discretization through optimized maximum likelihood	5	2	2	2	5	3
MI on symbol vectors with Equal Width discretization through optimized maximum likelihood	5	2	2	2	5	3
Pearson correlation coefficient	2	2	2	2	3	2
Qualitative distance measure	5	2	2	5	1	3
Spearman correlation coefficient	5	2	2	2	5	3
Symbol sequence similarity	5	2	2	2	5	3

Figure 10: Clustering (K-means) of base methods into 5 views (clusters) for the SynTReN *E. coli* dataset with 100 nodes and noise level 0.0. Each base method is a combination of a distance measure and a scoring scheme (cf. Supplementary File 1). The number (and the corresponding color) in a cell identifies the cluster to which a base method is assigned. Empty cells indicate that the corresponding base method has not been associated with any cluster. This can happen if the base method returns no interactions.

Measures	Scoring Schemes					
	ARACNE	AWE	AWE w/o TF	CLR	ID	MRNET
Dynamic Time Warping (Asymmetric constraint path)	2	2	2	2	2	2
Dynamic Time Warping (Symmetric constraint path 1)	2	2	2	2	2	2
Dynamic Time Warping (Symmetric constraint path 2)	2	2	2	2	2	2
Euclidian distance	2	2	2	2	2	2
Kendall correlation coefficient	3	2	2	2	3	2
L10Norm distance	2	2	2	2	1	2
Manhattan distance	2	2	2	2	2	2
Mean between symbolic sequence similarity and MI on symbol vectors with Equal Frequency discretization through optimized maximum likelihood	3	2	2	2	3	2
Mean between symbolic sequence similarity and MI on symbol vectors with Equal Width discretization through optimized maximum likelihood	3	2	2	2	3	2
MI - Equal Frequency discretization through optimized maximum likelihood	3	2	2	2	3	2
MI - Equal Frequency discretization through optimized maximum likelihood over coarse-grained variables (Kolmogorov-Sinai entropy)	1	2	2	2	1	2
MI - Equal Frequency discretization through shrinkage	3	2	2	2	3	2
MI - Equal Frequency discretization through shrinkage over coarse-grained variables (Kolmogorov-Sinai entropy)	1	2	2		1	2
MI - Equal Width discretization through optimized maximum likelihood	2	2	2	2	3	2
MI - Equal Width discretization through optimized maximum likelihood over coarse-grained variables (Kolmogorov-Sinai entropy)	2	2	2	2	1	2
MI - Equal Width discretization through shrinkage	2	2	2	2	3	2
MI - Equal Width discretization through shrinkage over coarse-grained variables (Kolmogorov-Sinai entropy)	1	2	2		1	2
MI on symbol vectors with Equal Frequency discretization through optimized maximum likelihood	3	2	2	2	3	2
MI on symbol vectors with Equal Width discretization through optimized maximum likelihood	3	2	2	2	3	2
Pearson correlation coefficient	2	2	2	2	2	2
Qualitative distance measure	3	2	2	3	1	2
Spearman correlation coefficient	3	2	2	2	3	2
Symbol sequence similarity	3	2	2	2	3	2

Figure 11: Clustering (K-means) of base methods into 3 views (clusters) for the SynTReN *Yeast* dataset with 100 nodes and noise level 0.0. Each base method is a combination of a distance measure and a scoring scheme (cf. Supplementary File 1). The number (and the corresponding color) in a cell identifies the cluster to which a base method is assigned. Empty cells indicate that the corresponding base method has not been associated with any cluster. This can happen if the base method returns no interactions.

Measures	Scoring Schemes					
	ARACNE	AWE	AWE w/o TF	CLR	ID	MRNET
Dynamic Time Warping (Asymmetric constraint path)	4	2	2	2	4	4
Dynamic Time Warping (Symmetric constraint path 1)	4	2	2	4	4	4
Dynamic Time Warping (Symmetric constraint path 2)	4	2	2	4	4	4
Euclidian distance	4	2	2	4	4	4
Kendall correlation coefficient	3	2	2	2	3	2
L10Norm distance	4	2	2	4	4	4
Manhattan distance	4	2	2	4	4	4
Mean between symbolic sequence similarity and MI on symbol vectors with Equal Frequency discretization through optimized maximum likelihood	3	2	2	2	3	2
Mean between symbolic sequence similarity and MI on symbol vectors with Equal Width discretization through optimized maximum likelihood	3	2	2	2	3	2
MI - Equal Frequency discretization through optimized maximum likelihood	3	2	2	2	3	2
MI - Equal Frequency discretization through optimized maximum likelihood over coarse-grained variables (Kolmogorov-Sinai entropy)	1	2	2	2	1	2
MI - Equal Frequency discretization through shrinkage	3	2	2	2	3	2
MI - Equal Frequency discretization through shrinkage over coarse-grained variables (Kolmogorov-Sinai entropy)	1	2	2		1	2
MI - Equal Width discretization through optimized maximum likelihood	2	2	2	2	3	2
MI - Equal Width discretization through optimized maximum likelihood over coarse-grained variables (Kolmogorov-Sinai entropy)	2	2	2	2	1	2
MI - Equal Width discretization through shrinkage	2	2	2	2	3	2
MI - Equal Width discretization through shrinkage over coarse-grained variables (Kolmogorov-Sinai entropy)	1	2	2		1	2
MI on symbol vectors with Equal Frequency discretization through optimized maximum likelihood	3	2	2	2	3	2
MI on symbol vectors with Equal Width discretization through optimized maximum likelihood	3	2	2	2	3	2
Pearson correlation coefficient	2	2	2	2	2	2
Qualitative distance measure	3	2	2	3	1	2
Spearman correlation coefficient	3	2	2	2	3	2
Symbol sequence similarity	3	2	2	2	3	2

Figure 12: Clustering (K-means) of base methods into 4 views (clusters) for the SynTReN *Yeast* dataset with 100 nodes and noise level 0.0. Each base method is a combination of a distance measure and a scoring scheme (cf. Supplementary File 1). The number (and the corresponding color) in a cell identifies the cluster to which a base method is assigned. Empty cells indicate that the corresponding base method has not been associated with any cluster. This can happen if the base method returns no interactions.

Measures	Scoring Schemes					
	ARACNE	AWE	AWE w/o TF	CLR	ID	MRNET
Dynamic Time Warping (Asymmetric constraint path)	4	2	2	2	4	4
Dynamic Time Warping (Symmetric constraint path 1)	4	2	2	4	4	4
Dynamic Time Warping (Symmetric constraint path 2)	4	2	2	4	4	4
Euclidian distance	4	2	2	4	4	4
Kendall correlation coefficient	5	2	2	2	5	3
L10Norm distance	4	2	2	4	4	4
Manhattan distance	4	2	2	4	4	4
Mean between symbolic sequence similarity and MI on symbol vectors with Equal Frequency discretization through optimized maximum likelihood	5	2	2	2	5	3
Mean between symbolic sequence similarity and MI on symbol vectors with Equal Width discretization through optimized maximum likelihood	5	2	2	2	5	3
MI - Equal Frequency discretization through optimized maximum likelihood	5	2	2	2	5	3
MI - Equal Frequency discretization through optimized maximum likelihood over coarse-grained variables (Kolmogorov-Sinai entropy)	1	2	2	2	1	2
MI - Equal Frequency discretization through shrinkage	5	2	2	2	5	3
MI - Equal Frequency discretization through shrinkage over coarse-grained variables (Kolmogorov-Sinai entropy)	1	2	2		1	2
MI - Equal Width discretization through optimized maximum likelihood	2	2	2	2	5	2
MI - Equal Width discretization through optimized maximum likelihood over coarse-grained variables (Kolmogorov-Sinai entropy)	2	2	2	2	1	2
MI - Equal Width discretization through shrinkage	2	2	2	2	5	2
MI - Equal Width discretization through shrinkage over coarse-grained variables (Kolmogorov-Sinai entropy)	1	2	2		1	2
MI on symbol vectors with Equal Frequency discretization through optimized maximum likelihood	5	2	2	2	5	3
MI on symbol vectors with Equal Width discretization through optimized maximum likelihood	5	2	2	2	5	3
Pearson correlation coefficient	2	2	2	2	2	2
Qualitative distance measure	5	2	2	5	1	3
Spearman correlation coefficient	5	2	2	2	5	3
Symbol sequence similarity	5	2	2	2	5	3

Figure 13: Clustering (K-means) of base methods into 5 views (clusters) for the SynTReN *Yeast* dataset with 100 nodes and noise level 0.0. Each base method is a combination of a distance measure and a scoring scheme (cf. Supplementary File 1). The number (and the corresponding color) in a cell identifies the cluster to which a base method is assigned. Empty cells indicate that the corresponding base method has not been associated with any cluster. This can happen if the base method returns no interactions.

D5InSilico - 3 Views

Methods	
Other5	1
Other2	1
Regression7	1
Meta2	2
Regression8	2
Meta4	2
Other4	2
Regression4	2
MutualInformation3	2
Regression5	2
Meta5	2
Regression6	2
MutualInformation4	2
Other6	2
Correlation2	2
MutualInformation2	2
Correlation1	2
Bayesian1	2
Regression3	2
Other1	2
Correlation3	2
Other3	2
Bayesian2	2
Regression1	2
Meta1	2
Bayesian6	3
Other8	3
Bayesian5	3
Meta3	3
Other7	3
Bayesian4	3
MutualInformation1	3
MutualInformation5	3
Regression2	3
Bayesian3	3

D5InSilico - 4 Views

Methods	
Other5	1
Other2	1
Regression7	1
Meta2	2
Correlation2	2
Correlation1	2
Meta4	2
Other4	2
Regression4	2
Correlation3	2
Bayesian6	3
Other8	3
Bayesian5	3
Meta3	3
Other7	3
Bayesian4	3
MutualInformation1	3
MutualInformation5	3
Regression2	3
Bayesian3	3
Regression8	4
MutualInformation3	4
Regression5	4
Meta5	4
Regression6	4
MutualInformation4	4
Other6	4
MutualInformation2	4
Bayesian1	4
Regression3	4
Other1	4
Other3	4
Bayesian2	4
Regression1	4
Meta1	4

D5InSilico - 5 Views

Methods	
Other5	1
Other2	1
Regression7	1
Other6	2
Meta2	3
Other8	3
Other7	3
Regression8	3
Meta4	3
Other4	3
Regression4	3
MutualInformation3	3
Regression5	3
Regression6	3
MutualInformation4	3
Bayesian6	3
Correlation2	3
MutualInformation2	3
Correlation1	3
Bayesian1	3
Regression3	3
Other1	3
Regression2	3
Correlation3	3
Other3	3
Bayesian2	3
Regression1	3
Meta1	3
Meta5	4
Bayesian5	5
Meta3	5
Bayesian4	5
MutualInformation1	5
MutualInformation5	5
Bayesian3	5

D5EColi - 3 Views

Methods	
Other2	1
Other5	1
Other6	2
Other3	3
Other4	3
MutualInformation5	3
Other8	3
Correlation1	3
Meta3	3
Bayesian6	3
MutualInformation2	3
Meta4	3
Other7	3
MutualInformation4	3
MutualInformation3	3
Meta5	3
Bayesian1	3
Bayesian2	3
Bayesian4	3
Bayesian5	3
Correlation2	3
Regression1	3
Bayesian3	3
Correlation3	3
Regression5	3
Regression4	3
Regression2	3
Regression7	3
Regression8	3
Regression3	3
Regression6	3
MutualInformation1	3
Meta2	3
Meta1	3
Other1	3

D5EColi - 4 Views

Methods	
Other2	1
Other5	1
Other6	2
Other3	3
Other4	3
MutualInformation5	3
Other8	3
Correlation1	3
Meta3	3
Bayesian6	3
MutualInformation2	3
Meta4	3
Other7	3
MutualInformation4	3
MutualInformation3	3
Bayesian1	3
Bayesian2	3
Bayesian4	3
Bayesian5	3
Correlation2	3
Regression1	3
Bayesian3	3
Correlation3	3
Regression5	3
Regression4	3
Regression2	3
Regression7	3
Regression8	3
Regression3	3
Regression6	3
MutualInformation1	3
Meta2	3
Meta1	3
Other1	3
Meta5	4

D5EColi - 5 Views

Methods	
Other2	1
Other5	1
Other6	2
Other3	3
Other4	3
MutualInformation5	3
Other8	3
Correlation1	3
Meta3	3
Bayesian6	3
MutualInformation2	3
Meta4	3
Other7	3
MutualInformation4	3
MutualInformation3	3
Bayesian1	3
Bayesian2	3
Bayesian4	3
Bayesian5	3
Correlation2	3
Regression1	3
Bayesian3	3
Correlation3	3
Regression5	3
Regression2	3
Regression7	3
Regression8	3
Regression3	3
Regression6	3
MutualInformation1	3
Meta2	3
Meta1	3
Other1	3
Meta5	4
Regression4	5

D5Yeast - 3 Views

Methods	
Regression2	1
Other2	1
Meta3	1
Other6	2
Other8	3
Meta1	3
Meta2	3
Regression1	3
Regression4	3
Regression3	3
Regression8	3
Bayesian5	3
Regression7	3
Bayesian6	3
Regression5	3
Regression6	3
Bayesian1	3
Bayesian4	3
Bayesian3	3
Bayesian2	3
Correlation3	3
MutualInformation5	3
Other1	3
MutualInformation4	3
Correlation2	3
MutualInformation2	3
Correlation1	3
Other4	3
MutualInformation1	3
Other3	3
Meta4	3
Other7	3
Other5	3
MutualInformation3	3
Meta5	3

D5Yeast - 4 Views

Methods	
Regression2	1
Other2	1
Meta3	1
Other6	2
Other8	3
Meta1	3
Meta2	3
Regression1	3
Regression4	3
Regression3	3
Regression8	3
Bayesian5	3
Regression7	3
Bayesian6	3
Regression5	3
Regression6	3
Bayesian1	3
Bayesian4	3
Bayesian3	3
Bayesian2	3
Correlation3	3
MutualInformation5	3
Other1	3
MutualInformation4	3
Correlation2	3
MutualInformation2	3
Correlation1	3
Other4	3
MutualInformation1	3
Other3	3
Meta4	3
Other7	3
Other5	3
MutualInformation3	3
Meta5	4

D5Yeast - 5 Views

Methods	
Regression2	1
Other2	1
Meta3	1
Other6	2
Other8	3
Meta1	3
Meta2	3
Regression1	3
Regression3	3
Regression8	3
Bayesian5	3
Regression7	3
Bayesian6	3
Regression5	3
Regression6	3
Bayesian1	3
Bayesian4	3
Bayesian3	3
Bayesian2	3
Correlation3	3
MutualInformation5	3
Other1	3
MutualInformation4	3
Correlation2	3
MutualInformation2	3
Correlation1	3
Other4	3
MutualInformation1	3
Other3	3
Meta4	3
Other7	3
Other5	3
MutualInformation3	3
Meta5	4
Regression4	5

Figure 14: Clustering (K-means) of base methods for the DREAM5 datasets into 3, 4 and 5 views (clusters). The number (and the corresponding color) in a cell identifies the cluster to which a base method is assigned.
