# Supplementary material for: Semi-Supervised Multi-View Learning for Gene Network Reconstruction
Source: PLoS One. 2015 Dec 7;10(12):e0144031. doi: 10.1371/journal.pone.0144031 (PMC4671612; doi:10.1371/journal.pone.0144031)
Supplement: S2 Fig — (PDF) [file pone.0144031.s005.pdf]

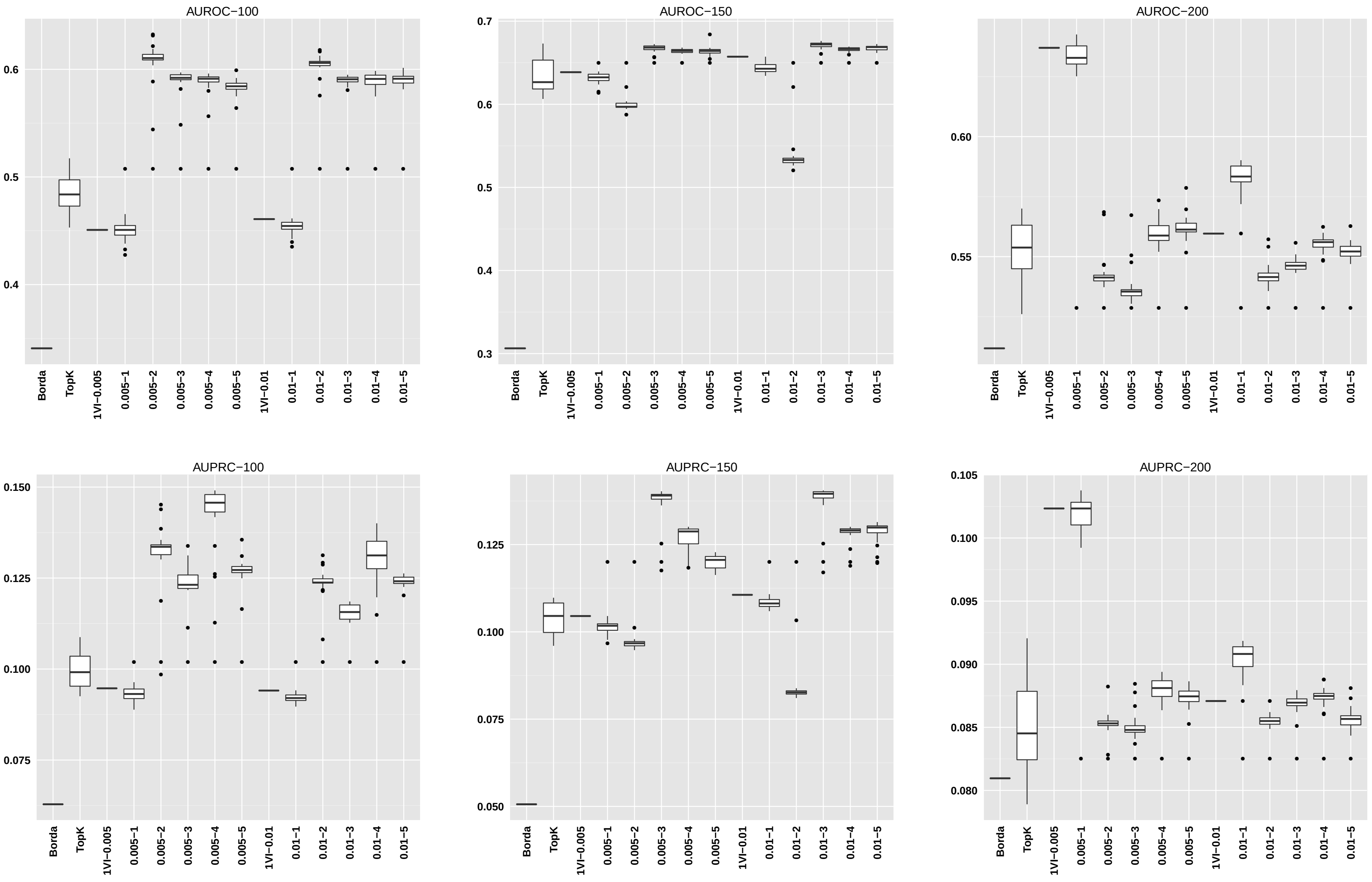

Figure 1: AUROC and AUPRC graphs for the SynTReN EColi dataset with noise level 0.0. The three columns of graphs represent results obtained with different numbers of genes in the dataset (100, 150 and 200, from left to right). Each graph represents box plots for (from left to right) Borda, TopK, 1VI with δ=0.005, GENERE with δ=0.005 and $V \in \{1, 2, 3, 4, 5\}$, 1VI with δ=0.01, GENERE with δ=0.01 and $V \in \{1, 2, 3, 4, 5\}$. The views are obtained with PCA. The boxplots for GENERE depict the distribution of AUROC/AUPRC values obtained by varying the number of iterations in [1,30]. The boxplots for TopK depict the distribution of AUROC/AUPRC obtained by varying the value of k in [1,20].

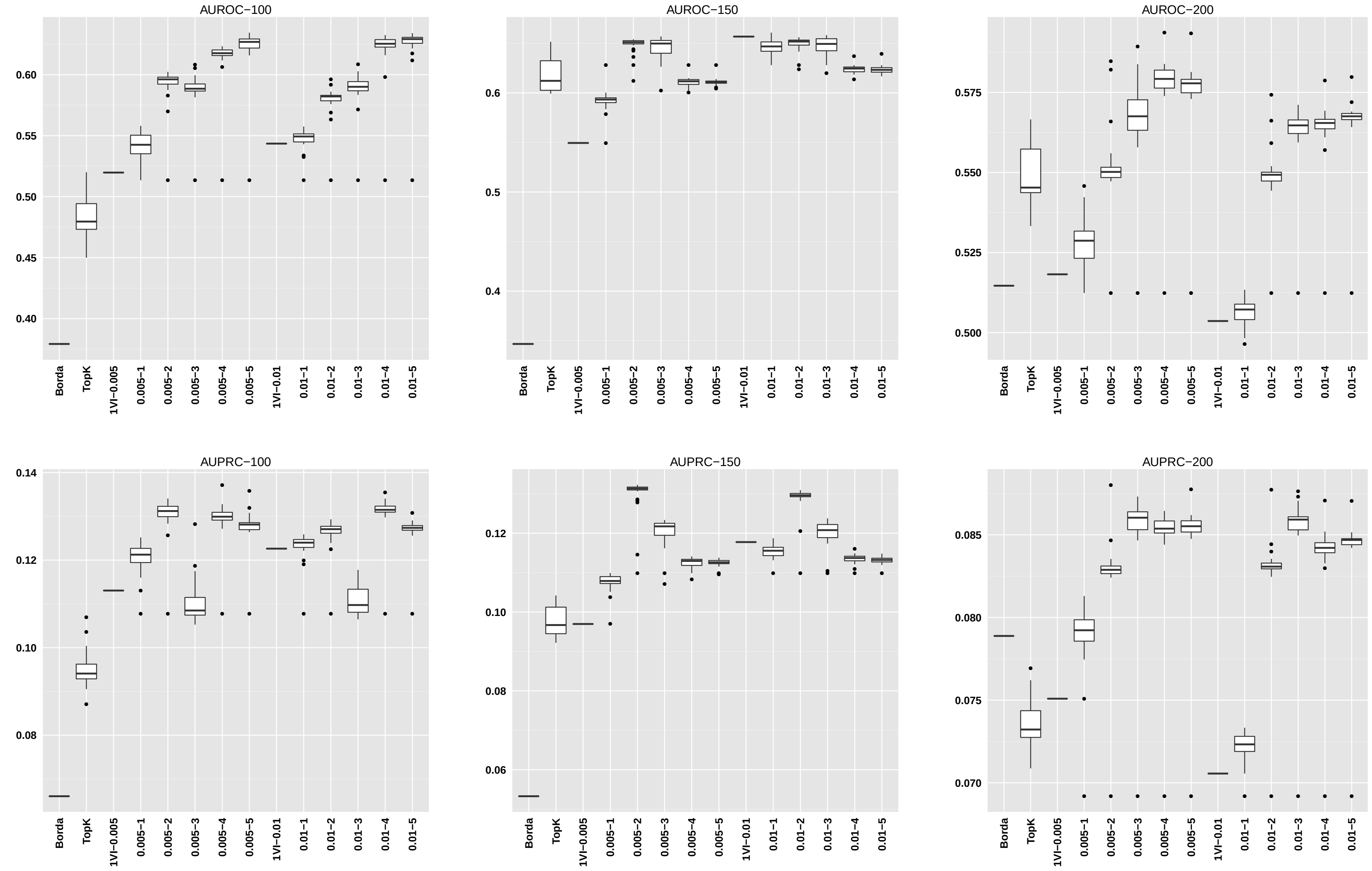

Figure 2: AUROC and AUPRC graphs for the SynTReN EColi dataset with noise level 0.1. The three columns of graphs represent results obtained with different numbers of genes in the dataset (100, 150 and 200, from left to right). Each graph represents box plots for (from left to right) Borda, TopK, 1VI with δ=0.005, GENERE with δ=0.005 and $V \in \{1, 2, 3, 4, 5\}$, 1VI with δ=0.01, GENERE with δ=0.01 and $V \in \{1, 2, 3, 4, 5\}$. The views are obtained with PCA. The boxplots for GENERE depict the distribution of AUROC/AUPRC values obtained by varying the number of iterations in [1,30]. The boxplots for TopK depict the distribution of AUROC/AUPRC obtained by varying the value of k in [1,20].

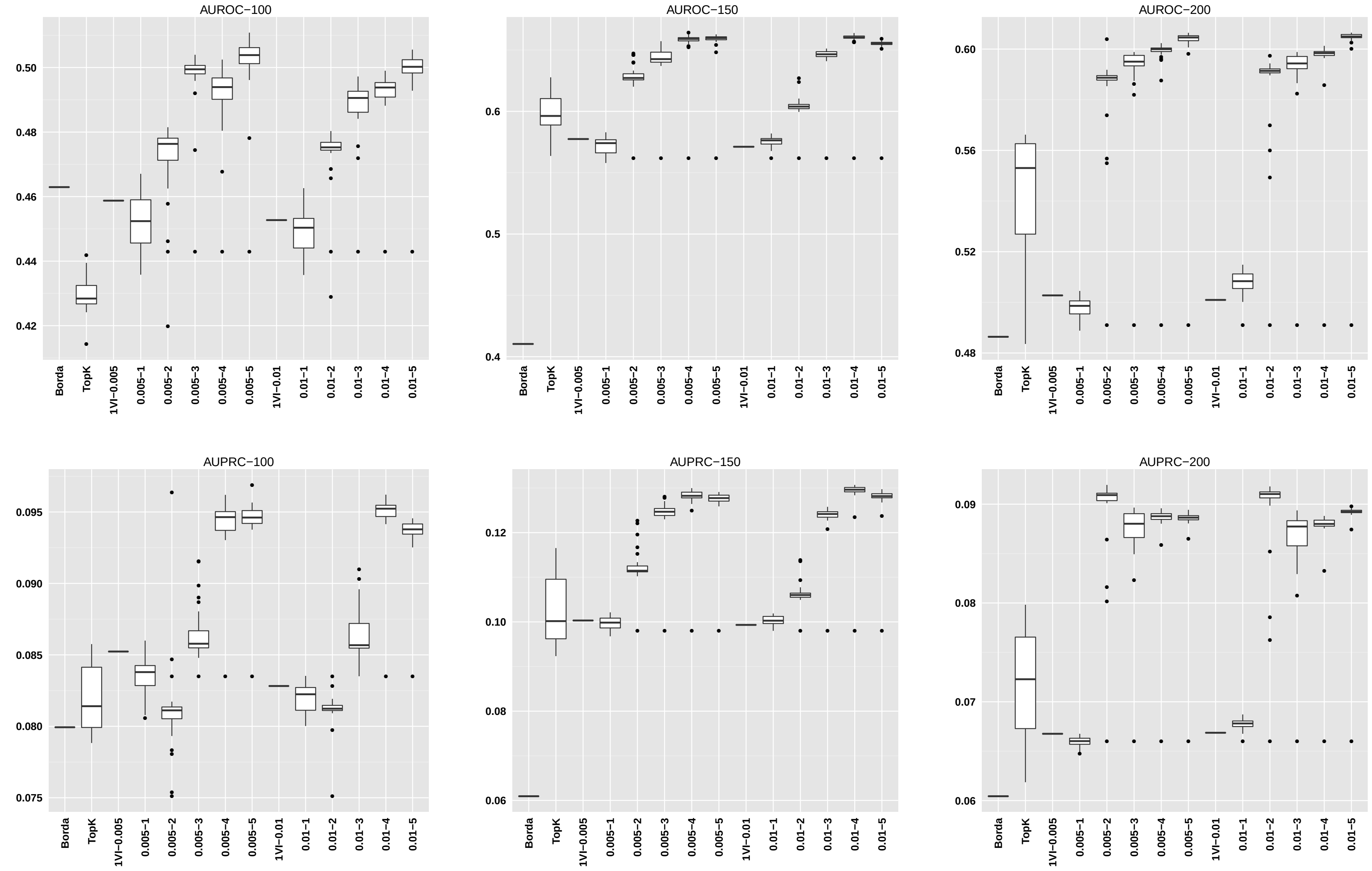

Figure 3: AUROC and AUPRC graphs for the SynTReN EColi dataset with noise level 0.5. The three columns of graphs represent results obtained with different numbers of genes in the dataset (100, 150 and 200, from left to right). Each graph represents box plots for (from left to right) Borda, TopK, 1VI with δ=0.005, GENERE with δ=0.005 and $V \in \{1, 2, 3, 4, 5\}$, 1VI with δ=0.01, GENERE with δ=0.01 and $V \in \{1, 2, 3, 4, 5\}$. The views are obtained with PCA. The boxplots for GENERE depict the distribution of AUROC/AUPRC values obtained by varying the number of iterations in [1,30]. The boxplots for TopK depict the distribution of AUROC/AUPRC obtained by varying the value of k in [1,20].

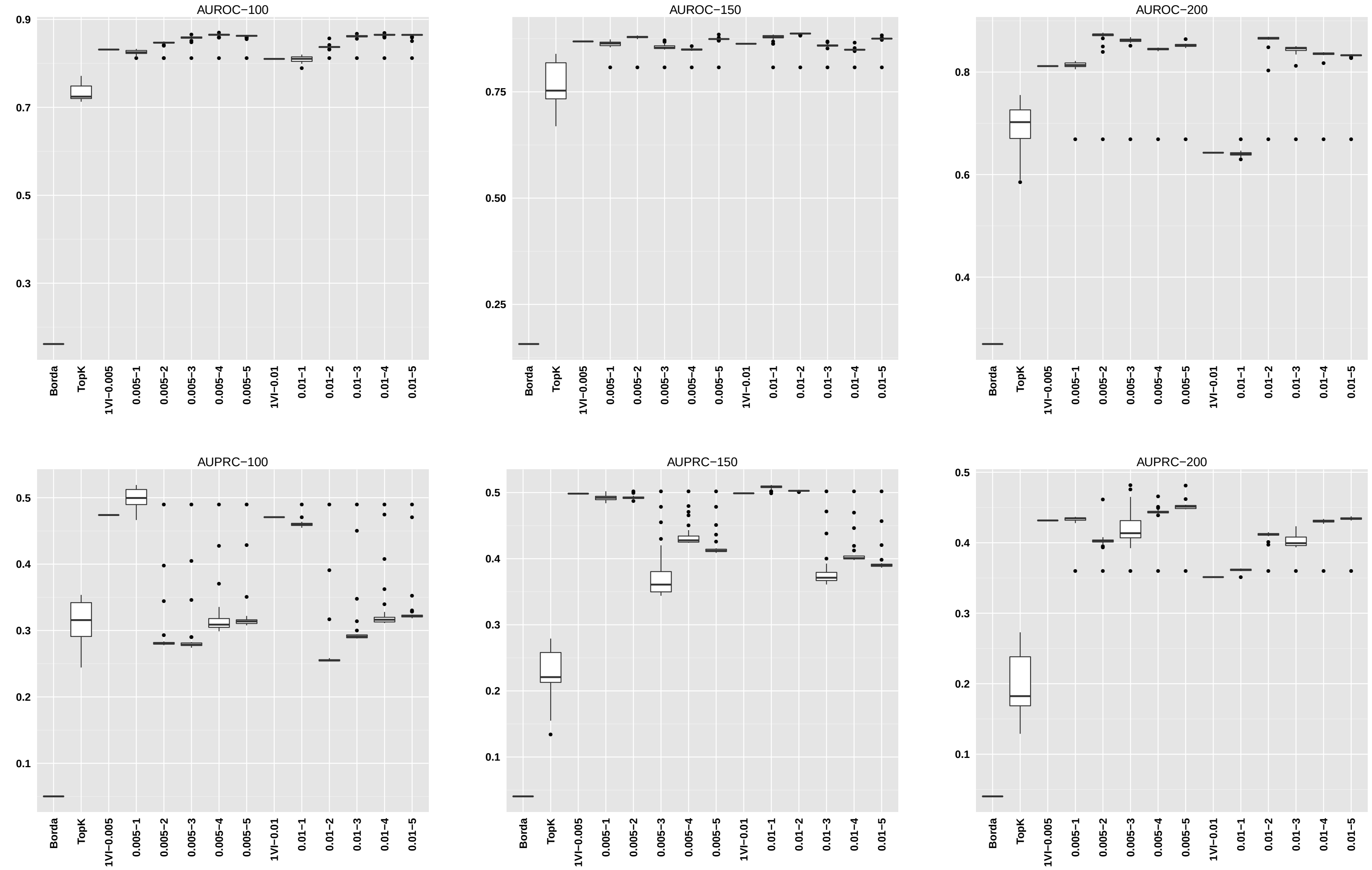

Figure 4: AUROC and AUPRC graphs for the SynTReN Yeast dataset with noise level 0.0. The three columns of graphs represent results obtained with different numbers of genes in the dataset (100, 150 and 200, from left to right). Each graph represents box plots for (from left to right) Borda, TopK, 1VI with δ=0.005, GENERE with δ=0.005 and $V \in \{1, 2, 3, 4, 5\}$, 1VI with δ=0.01, GENERE with δ=0.01 and $V \in \{1, 2, 3, 4, 5\}$. The views are obtained with PCA. The boxplots for GENERE depict the distribution of AUROC/AUPRC values obtained by varying the number of iterations in [1,30]. The boxplots for TopK depict the distribution of AUROC/AUPRC obtained by varying the value of k in [1,20].

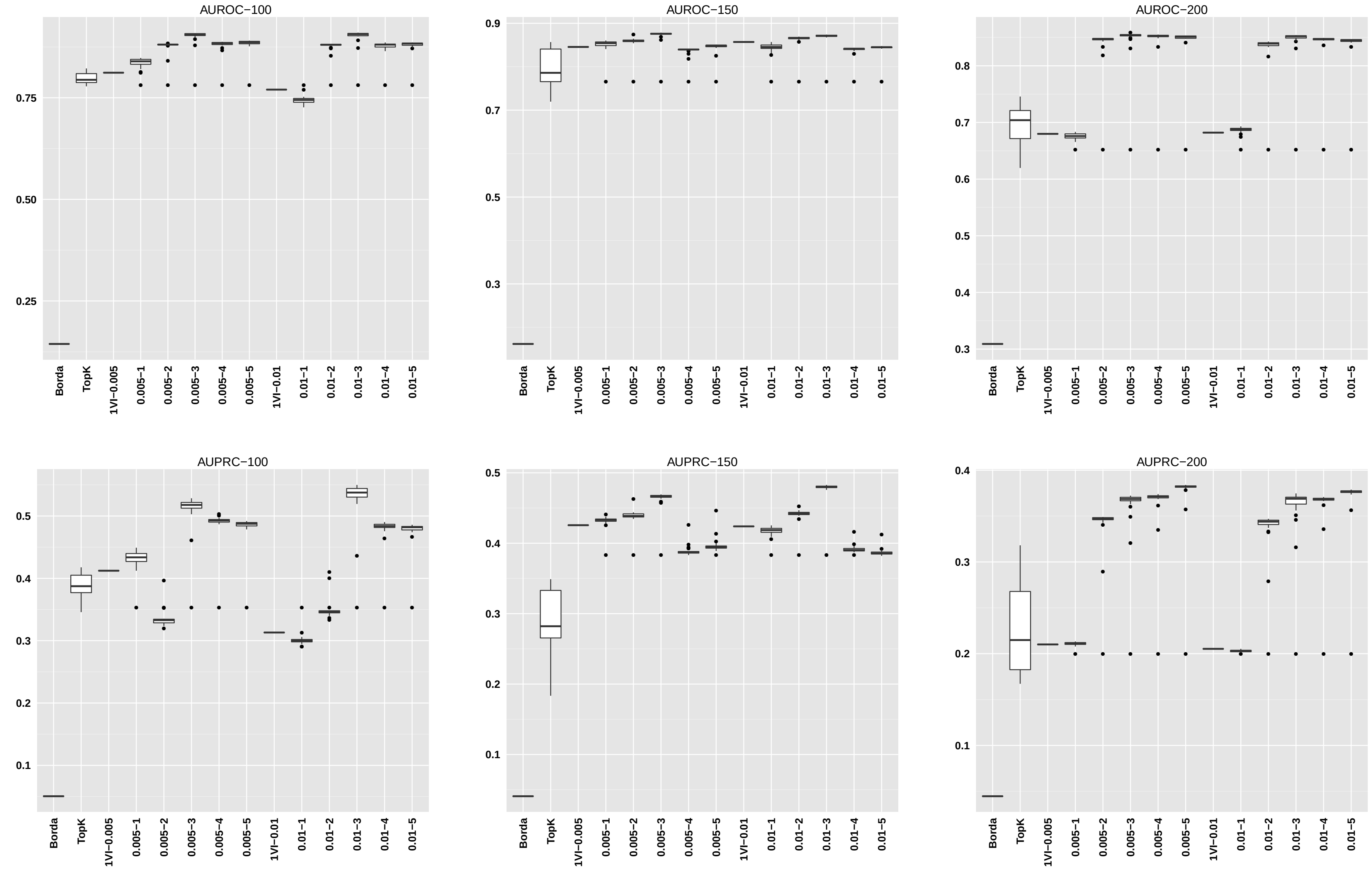

Figure 5: AUROC and AUPRC graphs for the SynTReN Yeast dataset with noise level 0.1. The three columns of graphs represent results obtained with different numbers of genes in the dataset (100, 150 and 200, from left to right). Each graph represents box plots for (from left to right) Borda, TopK, 1VI with δ=0.005, GENERE with δ=0.005 and $V \in \{1, 2, 3, 4, 5\}$, 1VI with δ=0.01, GENERE with δ=0.01 and $V \in \{1, 2, 3, 4, 5\}$. The views are obtained with PCA. The boxplots for GENERE depict the distribution of AUROC/AUPRC values obtained by varying the number of iterations in [1,30]. The boxplots for TopK depict the distribution of AUROC/AUPRC obtained by varying the value of k in [1,20].

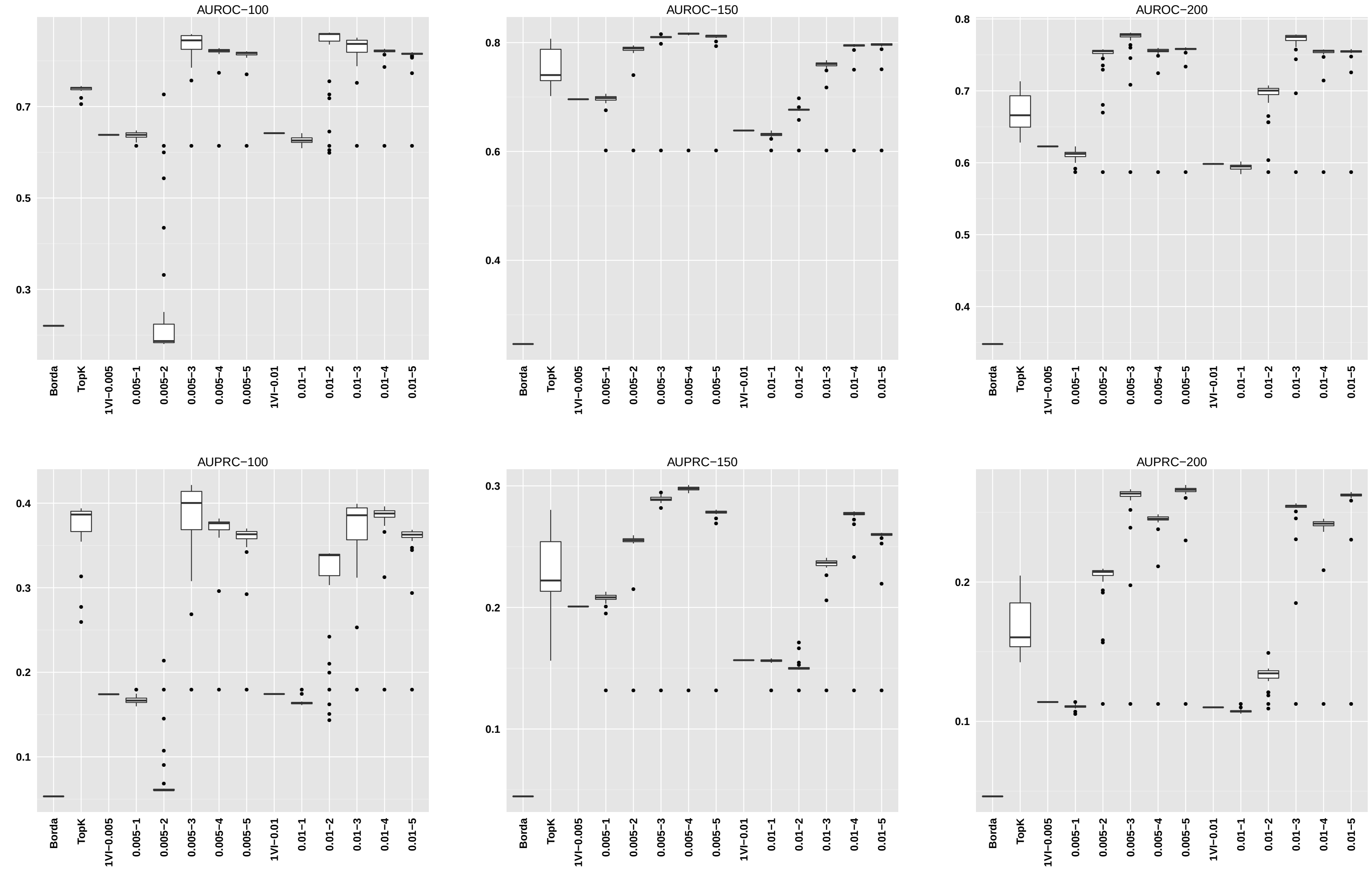

Figure 6: AUROC and AUPRC graphs for the SynTReN Yeast dataset with noise level 0.5. The three columns of graphs represent results obtained with different numbers of genes in the dataset (100, 150 and 200, from left to right). Each graph represents box plots for (from left to right) Borda, TopK, 1VI with δ=0.005, GENERE with δ=0.005 and $V \in \{1, 2, 3, 4, 5\}$, 1VI with δ=0.01, GENERE with δ=0.01 and $V \in \{1, 2, 3, 4, 5\}$. The views are obtained with PCA. The boxplots for GENERE depict the distribution of AUROC/AUPRC values obtained by varying the number of iterations in [1,30]. The boxplots for TopK depict the distribution of AUROC/AUPRC obtained by varying the value of k in [1,20].

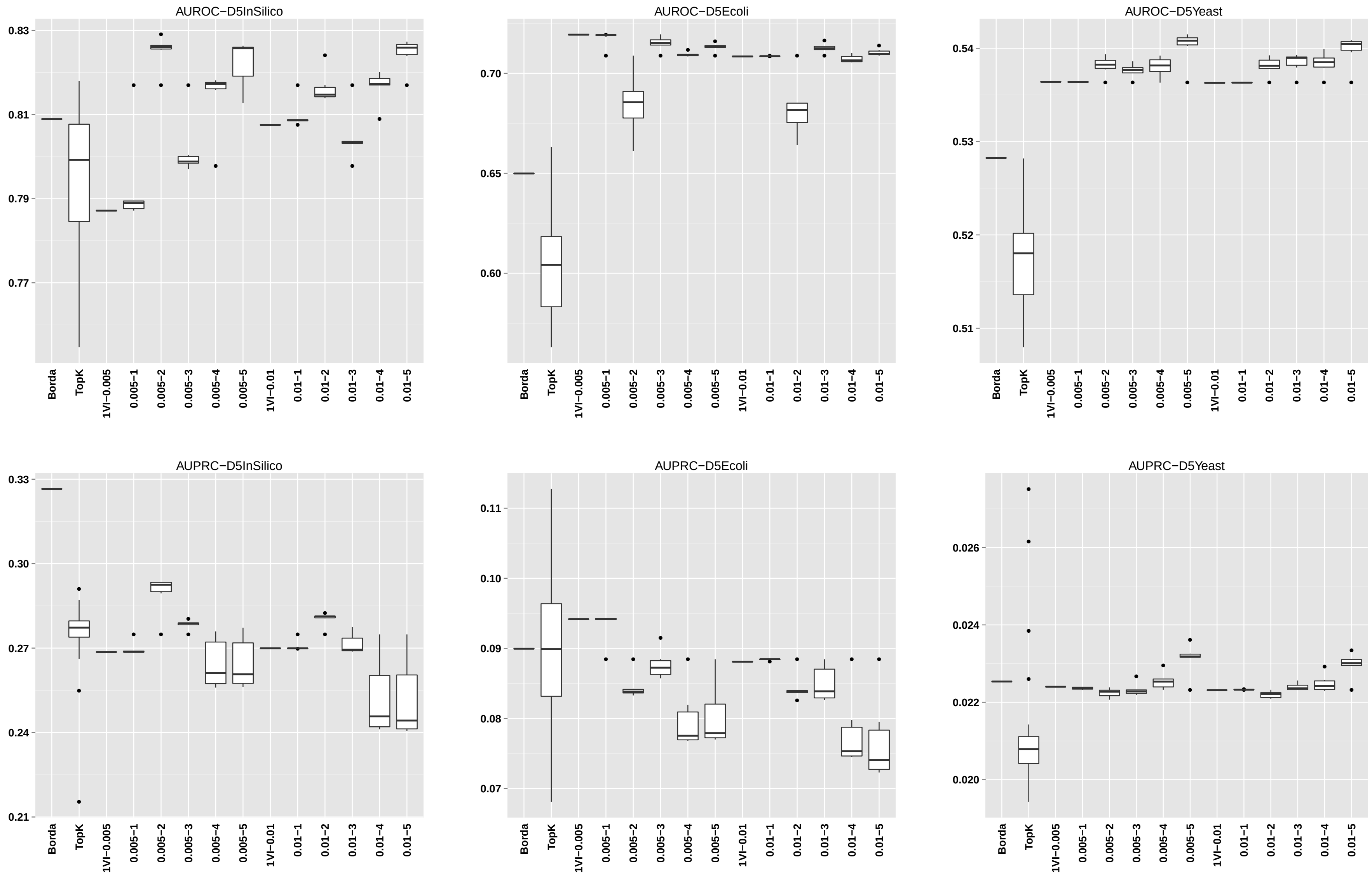

Figure 7: AUROC and AUPRC graphs for the DREAM5 datasets. The three columns of graphs represent results obtained on D5InSilico, D5EColi and D5Yeast, from left to right. Each graph represents box plots for (from left to right) Borda, TopK, 1VI with δ=0.005, GENERE with δ=0.005 and $V \in \{1, 2, 3, 4, 5\}$, 1VI with δ=0.01, GENERE with δ=0.01 and $V \in \{1, 2, 3, 4, 5\}$. The views are obtained with PCA. The boxplots for GENERE depict the distribution of AUROC/AUPRC values obtained by varying the number of iterations in [1,5]. The boxplots for TopK depict the distribution of AUROC/AUPRC obtained by varying the value of k in [1,20].
